# Comprehensive Studies of Adsorption Equilibrium and Kinetics for Selected Aromatic Organic Compounds on Activated Carbon

**DOI:** 10.3390/molecules29092038

**Published:** 2024-04-28

**Authors:** Małgorzata Wasilewska, Anna Derylo-Marczewska, Adam W. Marczewski

**Affiliations:** Department of Physical Chemistry, Institute of Chemical Sciences, Maria Curie-Sklodowska University, Maria Curie-Sklodowska Sq. 3, 20-031 Lublin, Poland; awmarcz@wp.pl

**Keywords:** adsorption of organic pollutants, phenol, nitrophenols, methylene blue, activated carbon, adsorption equilibrium and kinetics

## Abstract

This work presents a comprehensive analysis of the adsorption of selected aromatic organic compounds on activated carbons. Both the equilibrium and kinetics of adsorption were studied using UV–Vis spectrophotometry. The influence of a number of factors: pH, contact time, presence of an accompanying substance, adsorbate concentration, as well as the mass and size of adsorbent grains, on the adsorption process from aqueous solutions was investigated. Phenol, 2-nitrophenol, 3-nitrophenol, 4-nitrophenol and methylene blue (as an accompanying substance) were selected as adsorbates. GAC 1240W and RIAA activated carbons were used as adsorbents. The equilibrium data were analyzed using the generalized Langmuir isotherm equation (R^2^ = 0.912–0.996). Adsorption rate data were fitted using a multi-exponential kinetic equation (1 − R^2^ = (1.0 × 10^−6^)–(8.2 × 10^−4^)). As an additional parameter, the half-time was also used to present the influence of selected factors on the adsorption kinetics. An increase in the amount of adsorption was demonstrated with increasing contact time as well as with decreasing solution pH and adsorbent grain size. For selected systems, an increase in the adsorption rate was observed with increasing adsorbate concentration, adsorbent mass and at lower pH values. In some cases, the presence of an accompanying substance also resulted in an increase in adsorption kinetics. In the tested experimental systems, optimal conditions for adsorption were established (T = 298 K, pH = 2, contact time: 7 days, grain diameter: >0.5 mm and the ratio of the mass of the adsorbent to the volume of the adsorbate solution: 1 g/L). Additionally, the acid–base properties (potentiometric titration), morphology (SEM) and structure (TEM) of the used adsorbents were also examined.

## 1. Introduction

Toxic substances are chemical compounds that, upon contact, cause disruption of the function or death of living cells, organs or organisms. The degree of exposure to substances with toxic properties varies [1,2]. Among numerous substances of this type, aromatic organic compounds constitute a significant group. In the group of environmental pollutants, the most famous are undoubtedly phenols and their derivatives [3,4], dyes [5,6], pesticides [7,8] and pharmaceuticals [9,10]. Due to the interesting structure of the molecules of these compounds, they exhibit a number of important properties, as a result of which they exhibit several vital properties, resulting in their being widely used in various branches of industry. The release of toxic substances into the environment may cause periodic or permanent changes in the environment and loss of health or even life. It should be noted that they may have carcinogenic and mutagenic properties and are difficult to biodegrade [1,11]. Of course, it should also be added that some aromatic compounds do not directly exert a harmful effect on living organisms, but with long-term exposure they may have negative effects on the natural environment [9].

Due to the universality of applications and the greater or lesser toxicity of the compounds discussed, the most effective methods of purification of toxic substances are sought. For many years, a simple and environmentally neutral technology for eliminating toxic compounds has been the adsorption process using activated carbon. Due to the intensive development of industry, a significant increase in emissions of toxic substances, including aromatic organic compounds, has been observed in many countries around the world. The current state of the natural environment motivates scientists to constantly work on improving the effectiveness of adsorption methods. As is commonly known, a number of factors influence the efficiency of pollutant removal in both gas/solid and liquid/solid systems. The most important ones undoubtedly include the surface charge, structural and textural parameters of the adsorbent, the structure and hydrophobicity of the adsorbate and the conditions of the adsorption process, e.g., temperature [12,13,14], pH [15,16,17], mixing speed [18,19,20], the ratio of the mass of the adsorbent to the volume of the solution [21,22,23] or the presence of a competing substance [24,25,26]. These factors influence the adsorption effectiveness taking into account the adsorption uptake and rate. The equilibrium of adsorption process is well described for many different systems. However, the adsorption kinetics still needs extensive investigations, especially the influence of system characteristics and conductions of adsorption processes should be further analyzed.

The aim of this work was a comprehensive study of the adsorption of selected organic pollutants on activated carbon regarding the influence of various factors on process equilibrium and kinetics. In practical terms, this means that these studies can be used to determine optimal conditions for conducting the water and sewage treatment process using adsorption methods. The adsorption processes for phenol (F), 2-nitrophenol (2-NF), 3-nitrophenol (3-NF) and 4-nitrophenol (4-NF) as well as methylene blue (MB; used as an accompanying substance) on the commercial GAC 1240W (GAC) and RIAA activated carbons are analyzed. The above adsorbates, belonging to aromatic organic substances characterized by high toxicity, have numerous applications in industry. Moreover, all these substances dissolve relatively well in water and can therefore easily migrate in the environment, which poses a serious danger for plants, animals and humans. Phenol is currently mainly used to produce phenol–formaldehyde resins, medicines, detergents, herbicides, fungicides and dyes [27,28]. Unfortunately, this compound is highly toxic. Phenol exerts a negative effect immediately after ingestion, in contact with the skin or after inhalation. Additionally, it causes serious skin burns and eye damage. Moreover, it is also suspected of generating genetic defects and, through long-term or repeated exposure, damage to organs (nervous system, kidneys, liver) and is toxic to aquatic life, causing long-term effects [28,29]. 2-nitrophenol is used as an indicator and substrate in the production of dyes, paints, rubber chemicals and fungicides. Like phenol, 2-NF also has toxic properties. Inhalation of this compound causes irritation of the mucous membranes and coughing. When in contact with the skin and eyes, it causes irritation. Consumption of 2-nitrophenol results in irritation of the mucous membranes of the mouth, throat, esophagus and gastrointestinal tract and in large amounts leads to headache, methemoglobinemia, cardiac arrhythmia and spasm [30,31]. 3-nitrophenol is mainly used for the synthesis of dyes, drugs and is used as an indicator. 3-NF also is a toxic compound. When in contact with the skin, it causes irritation, and in contact with the eyes, it causes irritation of the mucous membranes. Ingestion of 3-nitrophenol results in headache, vomiting, ataxia, methemoglobinemia, cardiac arrhythmia, drop in blood pressure, spasms and shortness of breath [32,33,34]. 4-nitrophenol, currently, is used to synthesize paracetamol, phenethidine, acetophenetidine, indicators, fungicides, dyes, explosives and herbicides. 4-NF is also used in analytical chemistry as a pH indicator, which changes its color from colorless or light yellow to intense yellow. 4-nitrophenol also has toxic properties. This compound is harmful in contact with skin, if swallowed or inhaled. As a result of delayed interaction of 4-nitrophenol with the blood, it forms methemoglobin, which may cause kidney damage, anemia, skin and eye irritation, systemic poisoning, cyanosis, disorientation and loss of consciousness [35,36,37,38]. Methylene blue is widely used in the textile, food, paper, plastic, dyeing, cosmetics and pharmaceutical industries. Although this dye is not highly toxic, it can cause several harmful effects. In case of acute exposure, it causes increased heart rate, nausea, vomiting, shock, cyanosis, jaundice, and tissue necrosis in people [39,40,41,42].

Wasilewska and co-authors in [3] presented details of the study of the temperature dependencies of both the equilibrium and the adsorption kinetics of 2-, 3- and 4-nitrophenol on GAC activated carbon. Thermodynamic parameters were determined there, the adsorption mechanism was proposed and interactions in experimental systems were identified. A significant complement to the article [3] is this work, which presents the influence of other factors determining the efficiency of adsorption, mainly nitrophenols, from aqueous solutions on activated carbon. Thus, the influence of the adsorbent grain size and contact time on the adsorption rate of 2-, 3-, 4-nitrophenol was determined. Moreover, the dependence of the adsorption rate on the adsorbent mass and the initial adsorbate concentration was estimated for phenol and its nitro derivatives. Finally, the influences of pH (4-nitrophenol) and the presence of a competing substance (methylene blue, 2-, 3- and 4-nitrophenol) on both the equilibrium and the adsorption kinetics were also determined. The generalized Langmuir isotherm taking into account the energetic heterogeneity of adsorption system was used to describe the equilibrium data. Meanwhile, the adsorption kinetic data were analyzed using simple equations including first-order (FOE), second-order (SOE), mixed 1,2-order (MOE), fractal-like FOE (f-FOE), fractal-like SOE (f-SOE), fractal-like MOE (f-MOE) and multi-exponential (m-exp) kinetics equations. It should also be noted that the proposed methodology for testing the adsorption rate enabled frequent recording of concentration changes over time, resulting in at least 70 experimental points. Such a detailed scope of research together with exhaustive data analysis constitutes the basis for optimizing the adsorption process of aromatic organic compounds. And as we know, increasing the efficiency of a technological process even by a small percentage often makes the methodology used more economical and competitive with other proposed solutions.

## 2. Results

### 2.1. Adsorbent Characteristics

GAC 1240W (GAC) and RIAA activated carbons were selected as adsorbents. The textural properties of the above materials were estimated based on nitrogen adsorption/desorption measurements. Both carbons were characterized by a large specific surface area and a large pore volume with a micropore content of approximately 40–46% [3,4].

Additionally, the structural and morphological properties were also examined using transmission and scanning electron microscopy. Moreover, the acid–base properties were also investigated by potentiometric titration.

**Figure 1:**
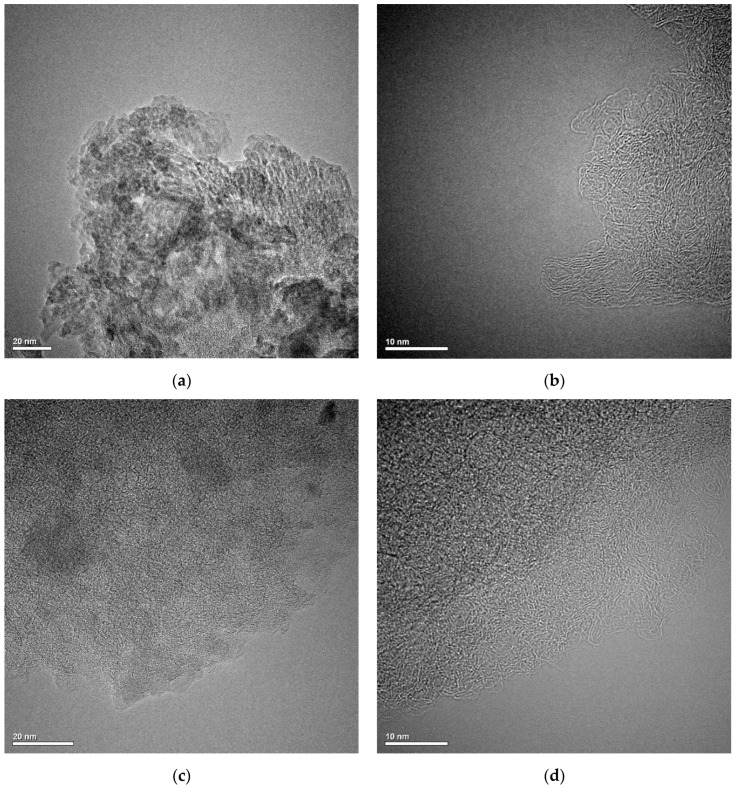
TEM micrographs of GAC (**a**,**b**) and RIAA (**c**,**d**) activated carbons.

Transmission electron microscopy was used to determine the structural properties of the tested carbon materials. Figure 1 shows TEM micrographs for GAC (Figure 1a,b) and RIAA (Figure 1c,d) activated carbons. As can be seen, these materials are characterized by a highly developed and disordered porous structure consisting mainly of tightly wound single layers of carbon.

**Figure 2:**
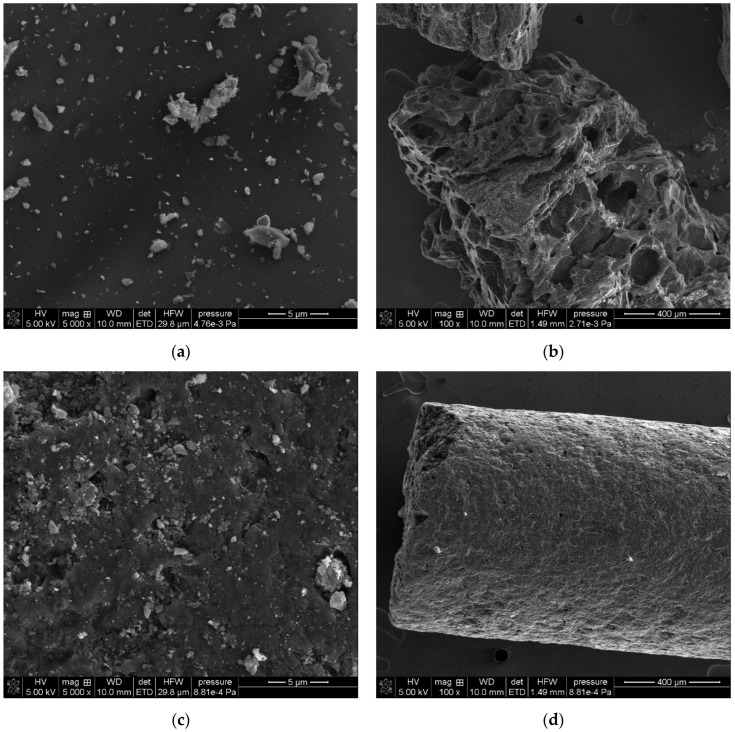
SEM micrographs of GAC 1240W (**a**,**b**) and RIAA (**c**,**d**) activated carbons.

Scanning electron microscopy was used to estimate the morphological properties of the carbon adsorbents used. Figure 2 presents SEM micrographs for GAC and RIAA materials. As can be seen, RIAA carbon granules have a cylindrical and regular shape. However, the grains of GAC material have a significantly different form—highly irregular. Additionally, based on SEM micrographs, it can be observed that the activated carbons used for research have a strongly expanded porous structure.

**Figure 3:**
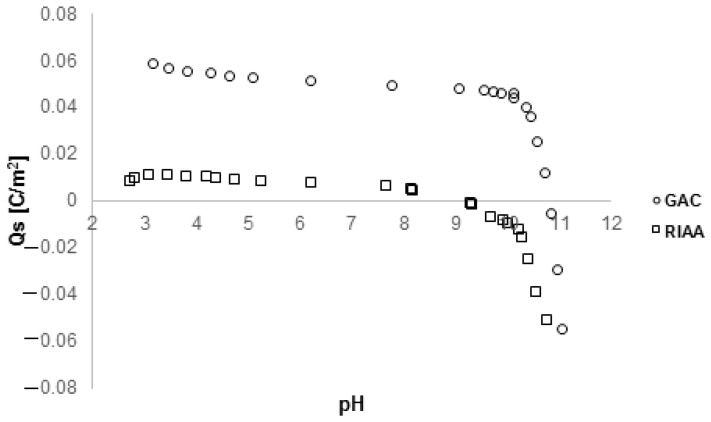
Surface charge density as a function of pH for GAC 1240W and RIAA activated carbons.

The acid–base properties of GAC and RIAA carbons were determined based on potentiometric titration measurements, which enabled the determination of the zero charge point pH_PZC_. Figure 3 shows the surface charge density as a function of pH. As can be seen, the pH_PZC_ values were 7.49 and 10.81 for RIAA and GAC carbons, respectively. This means that in the given experimental conditions, in most cases, the adsorbents had a small positive charge.

### 2.2. Adsorption Equilibrium and Kinetics

The influence of numerous factors (pH, contact time, presence of an accompanying substance, adsorbate concentration, as well as the mass and size of adsorbent grains) on the equilibrium and kinetics of adsorption of selected organic compounds was investigated. Equilibrium data were analyzed using the generalized Langmuir (GL) isotherm equation. The adsorption kinetics data were analyzed using first-order (FOE), second-order (SOE), mixed 1,2-order (MOE), fractal-like FOE (f-FOE), fractal-like SOE (f-SOE), fractal-like MOE (f-MOE) and multi-exponential (m-exp) kinetics equations.

#### 2.2.1. Effect of Adsorbent Grain Size

**Figure 4:**
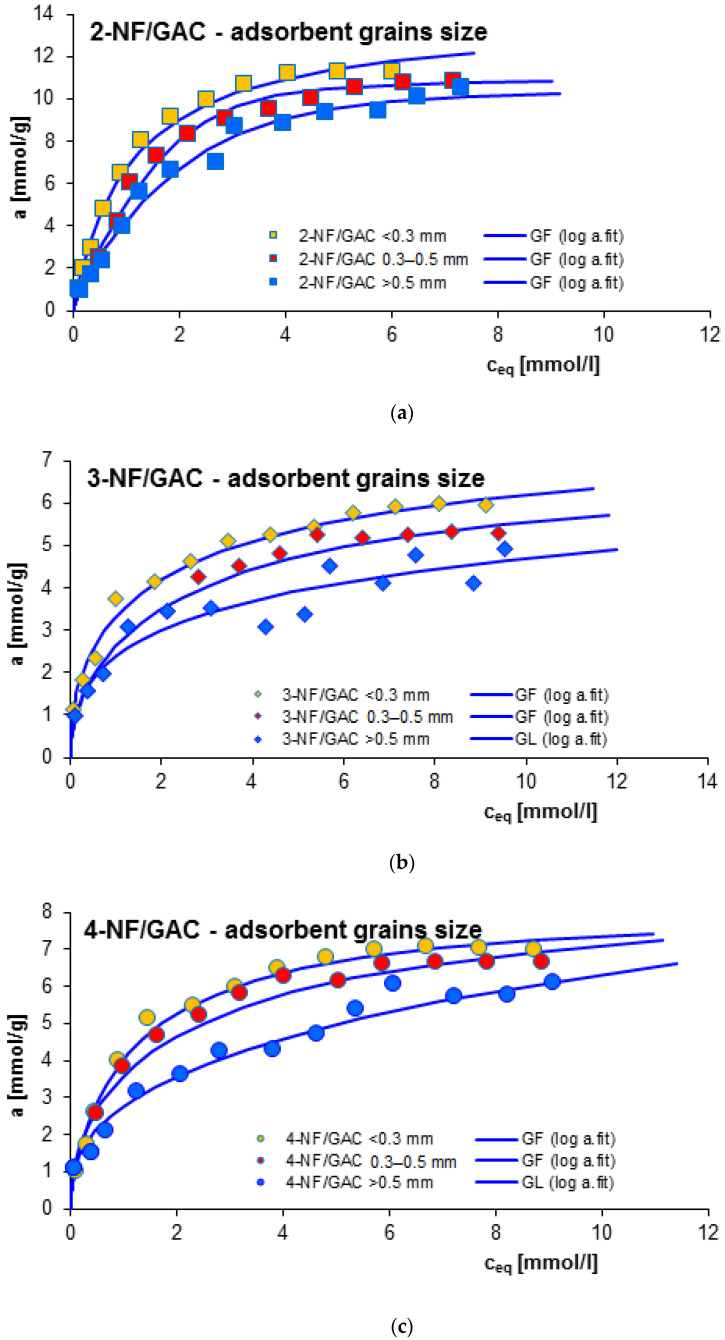
Comparison of adsorption isotherms for 2-NF (**a**), 3-NF (**b**) and 4-NF (**c**) on GAC activated carbon with different grain sizes: <0.3 mm, 0.3–0.5 mm and >0.5 mm.

One of the important factors in controlling the adsorption process is the size of the adsorbent grain. In general, the materials with a higher degree of fragmentation are characterized by enhanced adsorption effectiveness compared to adsorbents in the form of large granules. In this work, the influence of the adsorbent grain size was examined for adsorption of 2-, 3- and 4-nitrophenol (2-, 3- and 4-NF) on GAC 1240W (GAC) carbon. Three fractions of the above material were selected for testing with the following grain sizes: <0.3 mm, 0.3–0.5 mm and >0.5 mm. In Figure 4 the obtained adsorption isotherms are compared. As can be seen, a decrease in the adsorbed amount is observed with an increase in the size of the granules of the carbon used. The obtained maximum adsorption values were ~10.55–11.28 mmol/g, 4.91–5.94 mmol/g and 6.13–7.00 mmol/g for 2-NF, 3-NF and 4-NF, respectively. This effect is explained by the differentiated accessibility of adsorbent internal area (extended external surface for smaller grains) and divergent diffusion of adsorbate molecules (delay for larger grains) [43,44].

The equilibrium data in the different experimental systems were analyzed by using the generalized Langmuir (GL) isotherm equation, the parameters of which are listed in Table 1. As can be seen, for the systems 3-NF/GAC >0.5 mm and 4-NF/GAC >0.5 mm the adsorption equilibrium can be described by the full form of the GL isotherm. For the remaining cases, a simplified form—the generalized Freundlich (GF) isotherm—may be used. The values of the adjusted sorption capacities are 10.71–11.46 mmol/g, 5.13–6.02 mmol/g and 6.02–7.11 mmol/g for 2-NF, 3-NF and 4-NF, respectively. These results are in good agreement with the experimental adsorption values. The quality of the fit is very good, which is confirmed by the high values of the coefficients of determination (R^2^_2-NF_ = 0.988–0.996; R^2^_3-NF_ = 0.964–0.987; R^2^_4-NF_ = 0.954–0.987) and low values of standard deviations (SD(a)_2-NF_ = 0.019–0.028; SD(a)_3-NF_ = 0.025–0.051; SD(a)_4-NF_ = 0.028–0.053).

Although the highest adsorption efficiency was achieved for the systems with GAC activated carbon with a grain size of less than 0.3 mm, it should be remembered that dusty carbons are difficult to completely recover and reuse. Therefore, in the further part of the research, the fraction with a grain diameter above 0.5 mm was used.

#### 2.2.2. Effect of Contact Time

**Figure 5:**
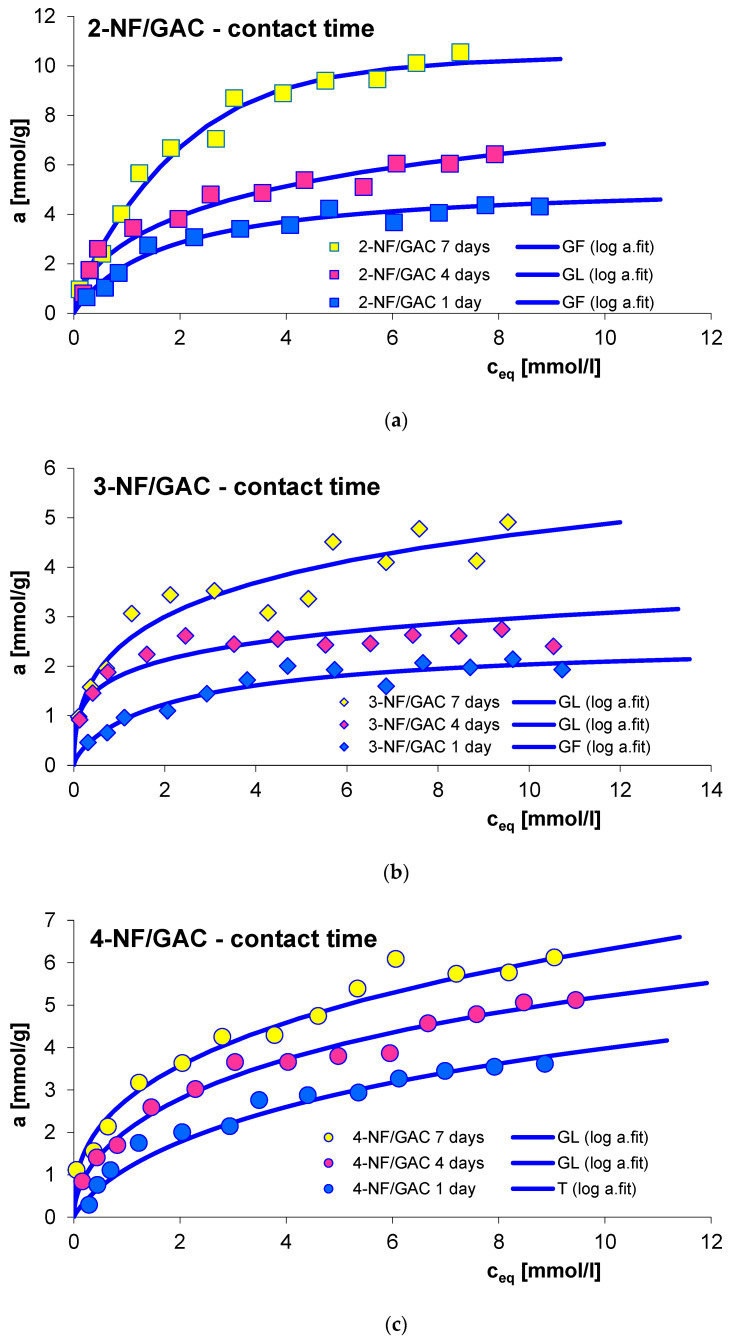
Comparison of adsorption isotherms of 2-NF (**a**), 3-NF (**b**) and 4-NF (**c**) on GAC activated carbon with different contact times: 1 day, 4 days and 7 days.

Another factor determining the adsorption effectiveness is the contact time of the adsorbate with the adsorbent. Generally, running the adsorption process for a longer period of time promotes the efficiency of this process. This effect was examined for adsorption of 2-, 3- and 4-nitrophenol (2-, 3- and 4-NF) on GAC 1240W (GAC) carbon. The adsorption processes were carried out for 1, 4 and 7 days. Figure 5 presents the obtained adsorption isotherms. As can be seen from the conducted research, an increase in the amount of adsorption was observed with the extension of the contact time of nitrophenols with GAC activated carbon. The obtained maximum adsorption values were 4.32–10.55 mmol/g, 2.14–4.91 mmol/g and 3.62–6.13 mmol/g for 2-NF, 3-NF and 4-NF, respectively. In the experimental systems tested, this effect was particularly visible, especially since the GAC material was characterized by a highly developed porous structure. Extending the contact time of the adsorbates allowed both adsorption on the surface active sites and penetration into the microstructure of the adsorbent [45,46].

The equilibrium data for experimental systems were described using the generalized Langmuir (GL) isotherm equation (the optimized parameters are listed in Table 2). As can be seen, the adsorption systems are well described by various forms of GL isotherm. The values of the optimized sorption capacities are 4.33–10.71 mmol/g, 2.01–5.13 mmol/g and 3.83–6.02 mmol/g for 2-NF, 3-NF and 4-NF, respectively. These values are comparable to experimental adsorption values. The quality of fit is very good, which is confirmed by high values of determination coefficients (R^2^_2-NF_ = 0.943–0.988; R^2^_3-NF_ = 0.912–0.964; R^2^_4-NF_ = 0.947–0.991) and low values of standard deviations (SD(a)_2-NF_ = 0.019–0.069; SD(a)_3-NF_ = 0.039–0.054; SD(a)_4-NF_ = 0.023–0.071).

Generally, the activated carbons used to remove contaminants from aqueous solutions are characterized by a highly developed porous structure, which allows adsorption on the surface as well as penetration into the interior of the adsorbent grains. Therefore, in the further part of the study, the adsorption process was carried out for 7 days.

#### 2.2.3. Effect of Adsorbent Mass

**Figure 6:**
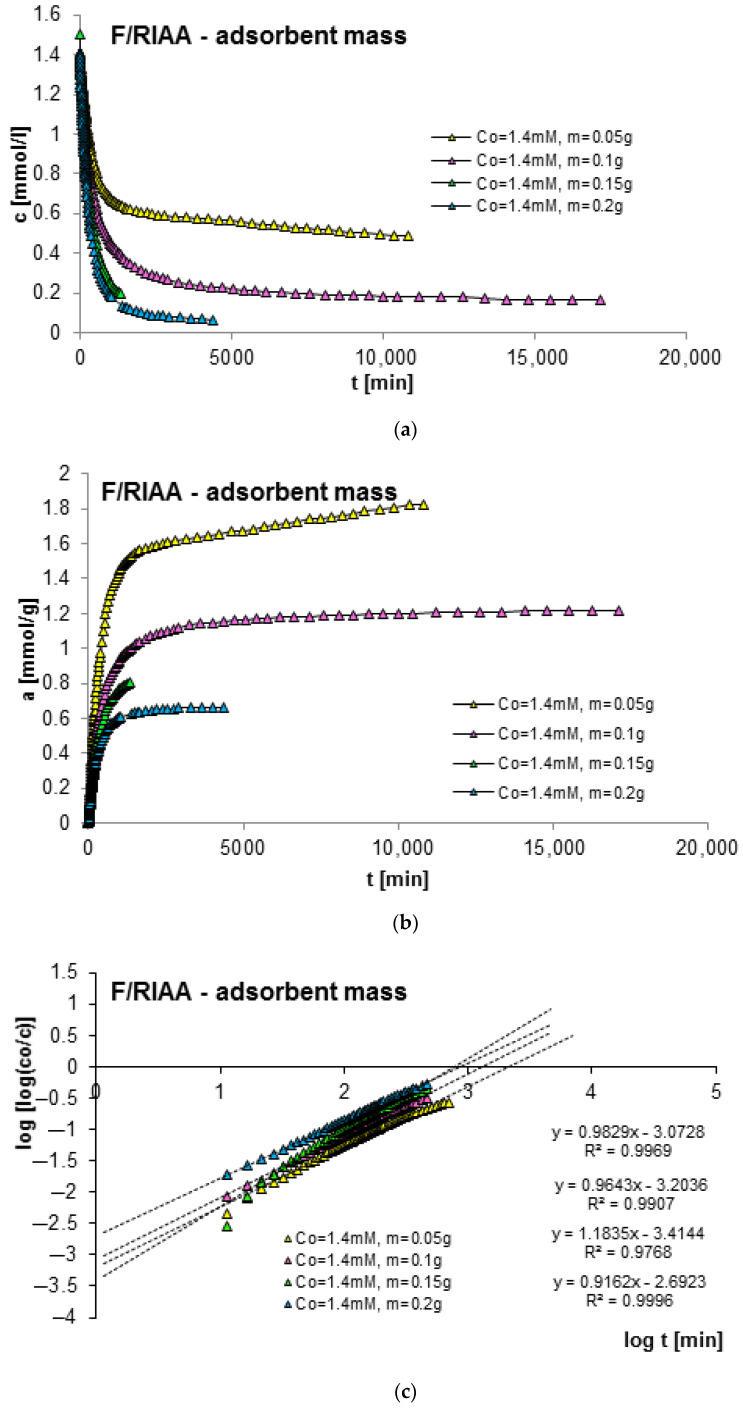
Comparison of the kinetics of adsorption of phenol (F) on RIAA activated carbon at a constant initial adsorbate concentration and variable adsorbent mass presented in the profile of concentration changes over time (**a**), relative adsorption over time (**b**) and in Bangham’s linear coordinates (**c**).

**Figure 7:**
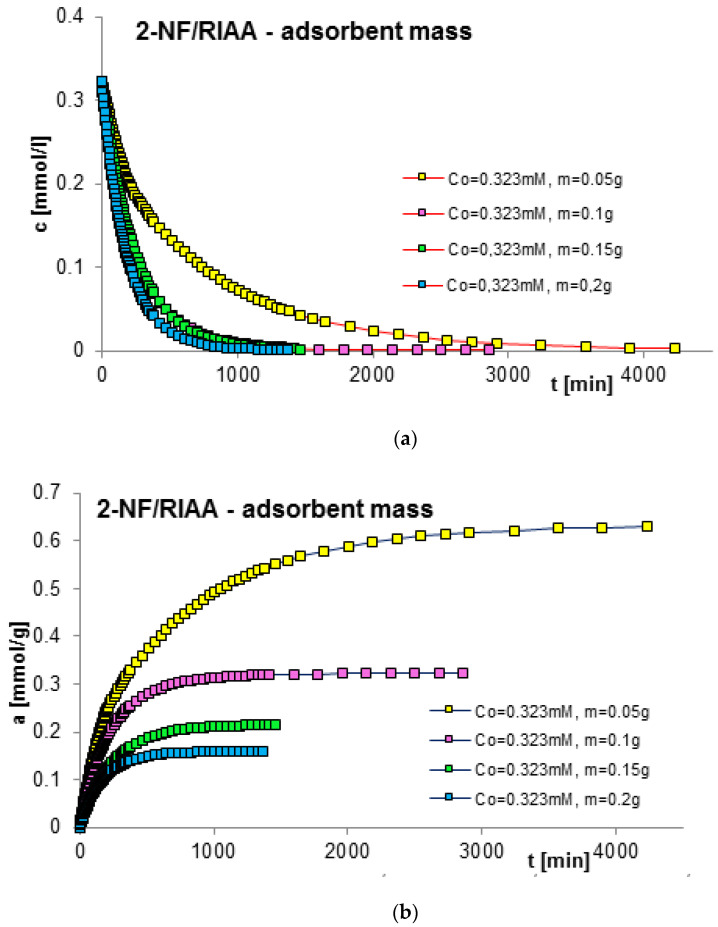

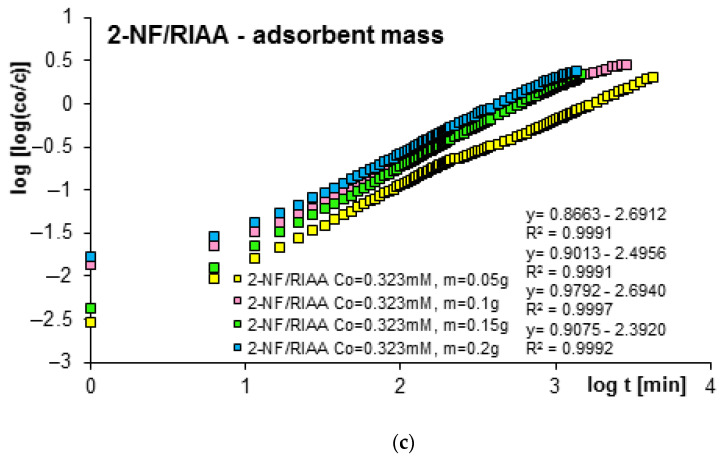
Comparison of the kinetics of adsorption of 2-nitrophenol (2-NF) on RIAA activated carbon at a constant initial adsorbate concentration and variable adsorbent mass presented in the profile of concentration changes over time (**a**), relative adsorption over time (**b**) and in Bangham’s linear coordinates (**c**).

**Figure 8:**
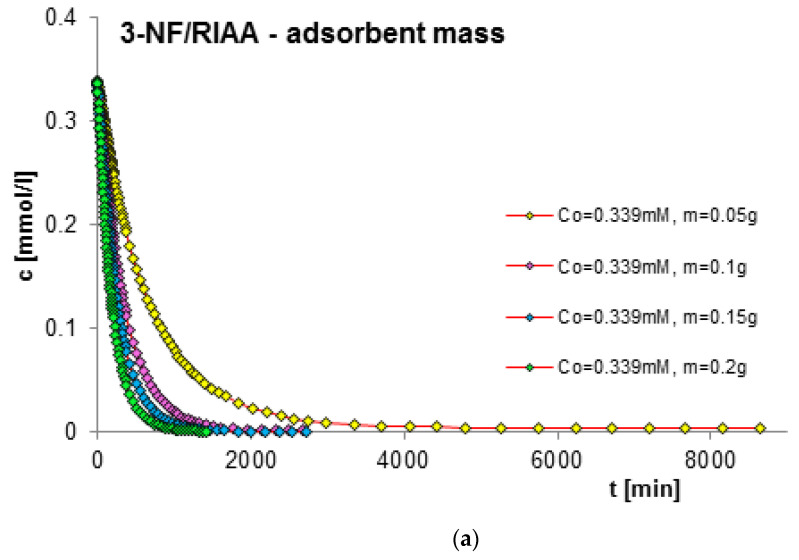

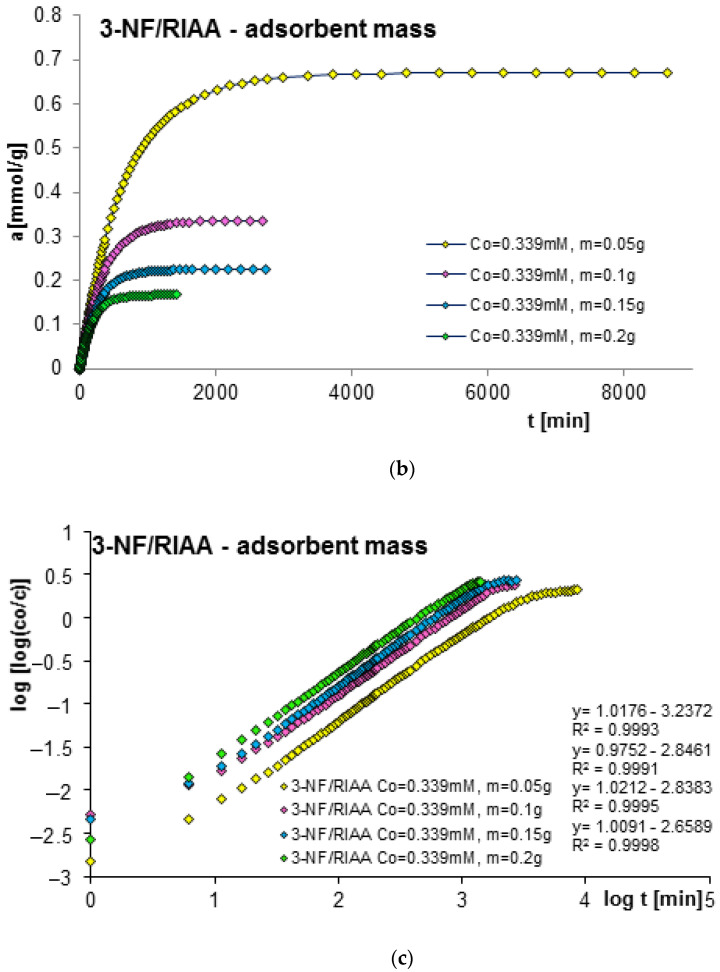
Comparison of the kinetics of adsorption of 3-nitrophenol (3-NF) on RIAA activated carbon at a constant initial adsorbate concentration and variable adsorbent mass presented in the profile of concentration changes over time (**a**), relative adsorption over time (**b**) and in Bangham’s linear coordinates (**c**).

**Figure 9:**
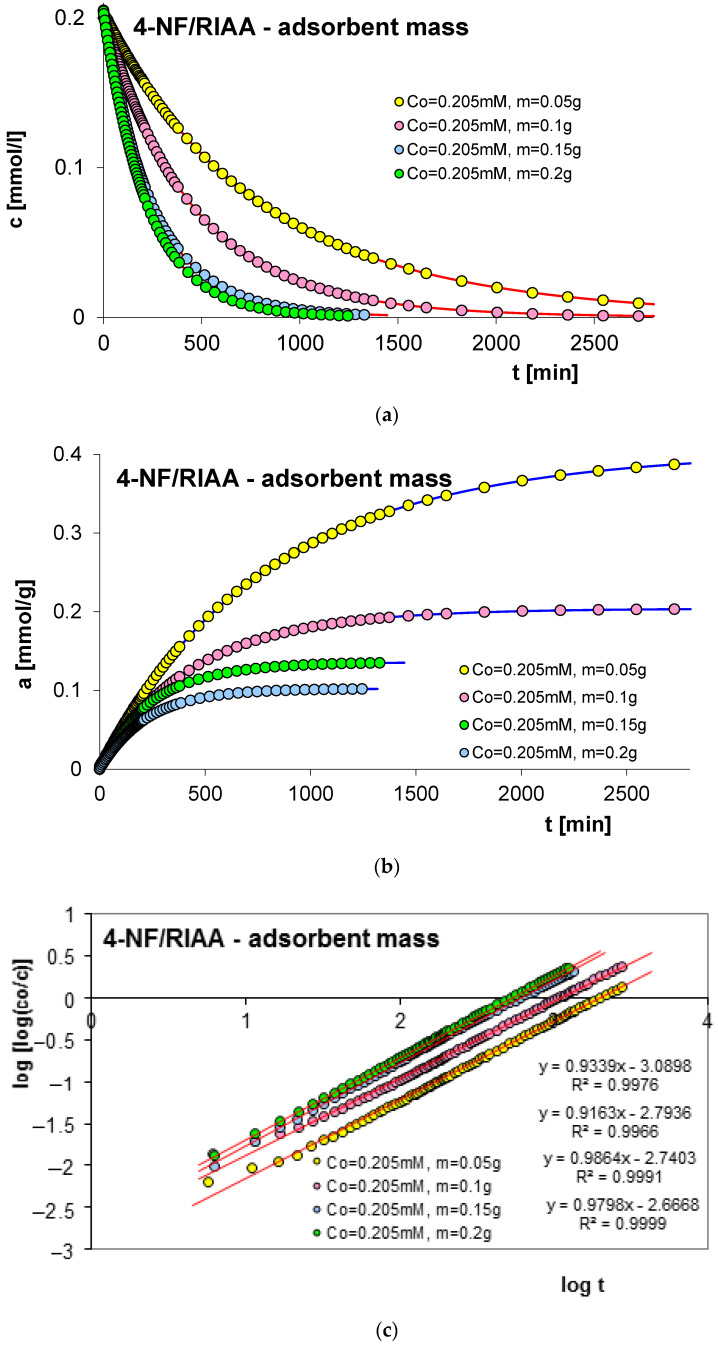
Comparison of the kinetics of adsorption of 4-nitrophenol (4-NF) on RIAA activated carbon at a constant initial adsorbate concentration and variable adsorbent mass presented in the profile of concentration changes over time (**a**), relative adsorption over time (**b**) and in Bangham’s linear coordinates (**c**).

The next factor determining the effectiveness of the adsorption process with regard to adsorption equilibrium and kinetics is the mass of the adsorbent. In order to estimate its impact on adsorption kinetics, measurements of concentration profiles for phenol (F) as well as for 2-, 3- and 4-nitrophenol (2-, 3- and 4-NF) adsorption were carried out on RIAA activated carbon. The kinetics measurements were carried out in the following conditions: constant initial concentration of the adsorbate (c_0 F_ = 1.4 mM; c_0 2-NF_ = 0.323 mM; c_0 3-NF_ = 0.339 mM, c_0 4-NF_ = 0.205 mM) and variable adsorbent mass (0.05; 0.1; 0.15; 0.2 g). Figure 6, Figure 7, Figure 8 and Figure 9 present a comparison of the adsorption kinetics of F (Figure 6), 2-NF (Figure 7), 3-NF (Figure 8) and 4-NF (Figure 9) on RIAA presented in the profile of concentration changes over time (Figure 6a, Figure 7a, Figure 8a and Figure 9a), relative adsorption in time (Figure 6b, Figure 7b, Figure 8b and Figure 9b) and in linear Bangham coordinates (Figure 6c, Figure 7c, Figure 8c and Figure 9c).

A clear influence of adsorbent mass on the process kinetics is observed. As the mass of the adsorbent increases, the rate of adsorption of the tested organic compounds increases. This effect can be attributed to the increased surface area of the adsorbent and the availability of active sites [47,48]. Additionally, it was noticed that for all experimental systems the initial adsorption rate of phenol and its derivatives is proportional to the mass of the adsorbent, while the amount of adsorption per unit mass of the adsorbate decreases with the increase in the amount of adsorbent. Based on the data analysis, it can also be concluded that the initial course of adsorption kinetics in the tested systems is linear.

The analysis of the kinetics of adsorption of selected organic compounds on RIAA activated carbon depending on the variable mass of the adsorbent began with the presentation of experimental data in linear coordinates of Bangham’s relations [49]. It was observed that the graphs are almost linear and their slopes are close to unity (p _F_: 0.92–1.18; p _2-NF_: 0.87–0.98, p _3-NF_: 0.98–1.02; p _4-NF_: 0.92–0.99), which excludes the possibility of using the IDM model (*p* = 0.5) to describe the kinetics of the tested experimental systems.

Therefore, in the further part of the kinetic data analysis, simple equations and models of adsorption kinetics were used, including FOE, SOE, MOE, f-FOE, f-SOE, f-MOE and m-exp. The relative standard deviations obtained for applied equations are listed in Table S1. As can be seen, for all experimental systems, the best quality of fit was obtained using a multi-exponential equation (SD(c)/c_o_ = 0.086–0.832%), the parameters of which are summarized in Table 3.

The analysis of the parameter values contained in Table 3 indicates that the adsorption kinetics in the tested experimental systems can be described using one, two or three terms of a multi-exponential equation. At the same time, it should be noted that for systems with three terms, one of them has a very small value. The determined half-times of adsorption kinetics in the tested experimental systems decrease with the increase in the mass of the adsorbent, which confirms the increase in the adsorption rate in systems with larger RIAA carbon mass (higher availability of adsorbent). The quality of the fit is very good, which can be confirmed by the low values of the relative standard deviations (SD(c)/c_0 F_: 0.094–0.832%; SD(c)/c_0 2-NF_: 0.251–0.512%; SD(c)/c_0 3-NF_: 0.231–0.480%; SD(c)/c_0 4-NF_: 0.086–0.327%) and the indeterminacy coefficient (1 − R^2^_F_: 8.5 × 10^−6^–8.2 × 10^−4^; 1 − R^2^_2-NF_: 5.6 × 10^−5^–2.6 × 10^−4^; 1 − R^2^_3-NF_: 4.5 × 10^−5^–2.0 × 10^−4^; 1 − R^2^_4-NF_: 7.7 × 10^−6^–9.9 × 10^−5^).

#### 2.2.4. Effect of Adsorbate Concentrations

**Figure 10:**
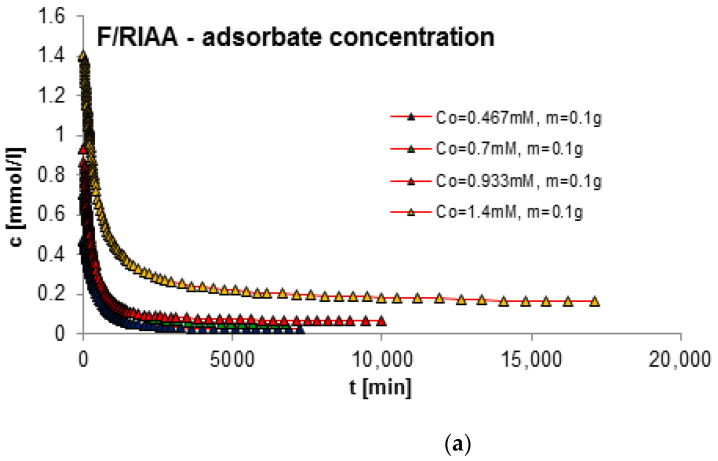

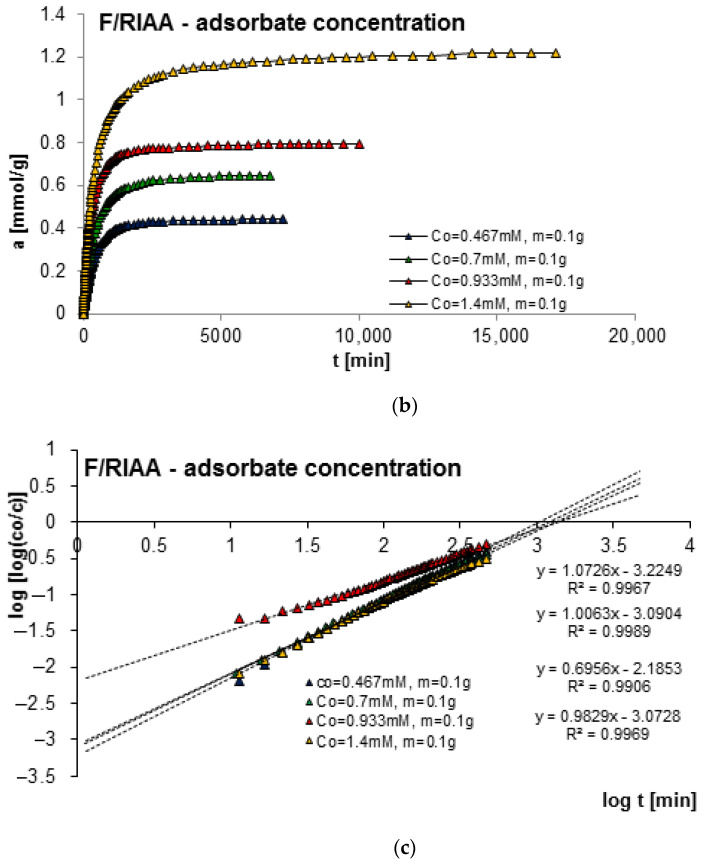
Comparison of the adsorption kinetics of F on RIAA activated carbon with constant adsorbent mass and variable initial adsorbate concentration presented in the profile of concentration changes over time (**a**), relative adsorption over time (**b**) and in Bangham’s linear coordinates (**c**).

**Figure 11:**
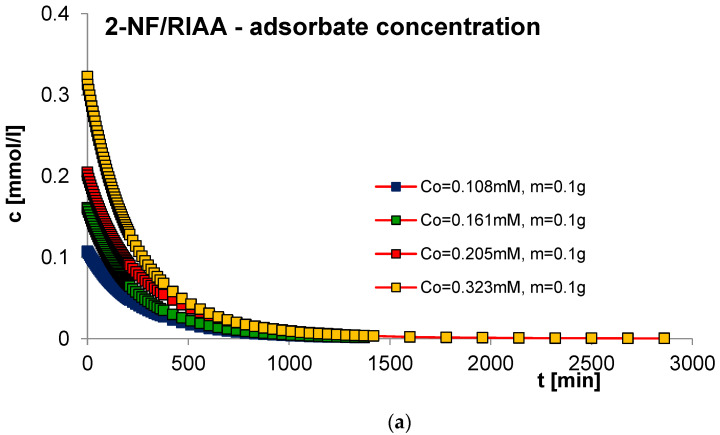

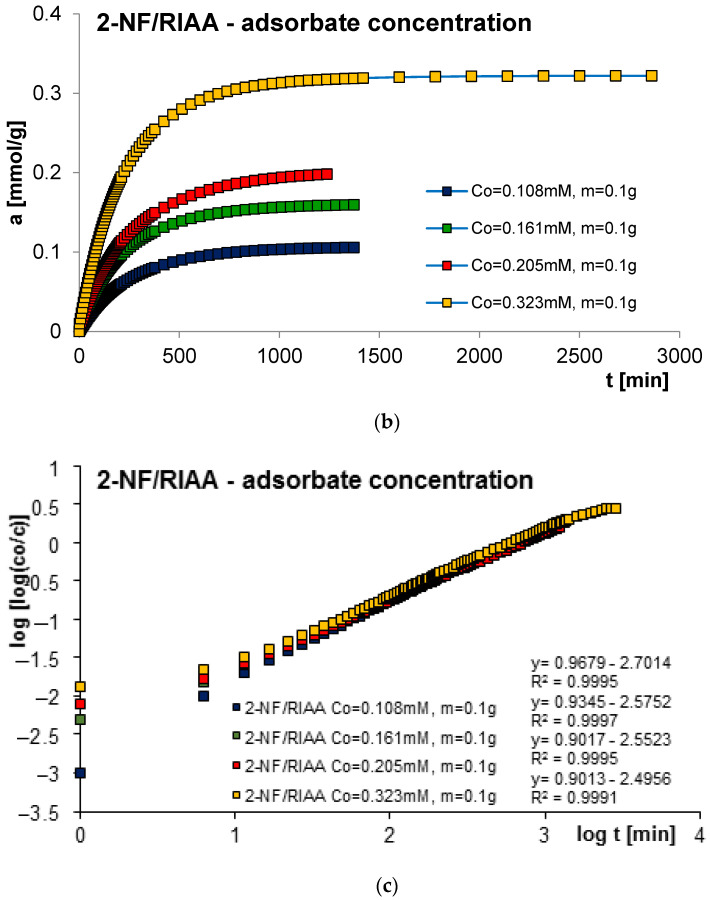
Comparison of the adsorption kinetics of 2-NF on RIAA activated carbon with constant adsorbent mass and variable initial adsorbate concentration presented in the profile of concentration changes over time (**a**), relative adsorption over time (**b**) and in Bangham’s linear coordinates (**c**).

**Figure 12:**
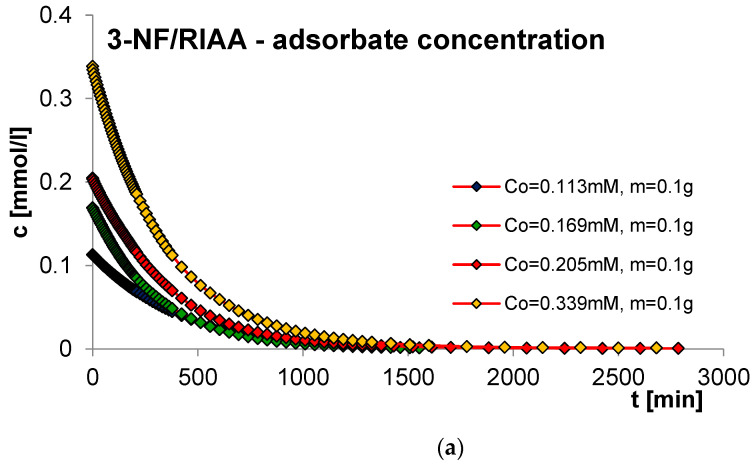

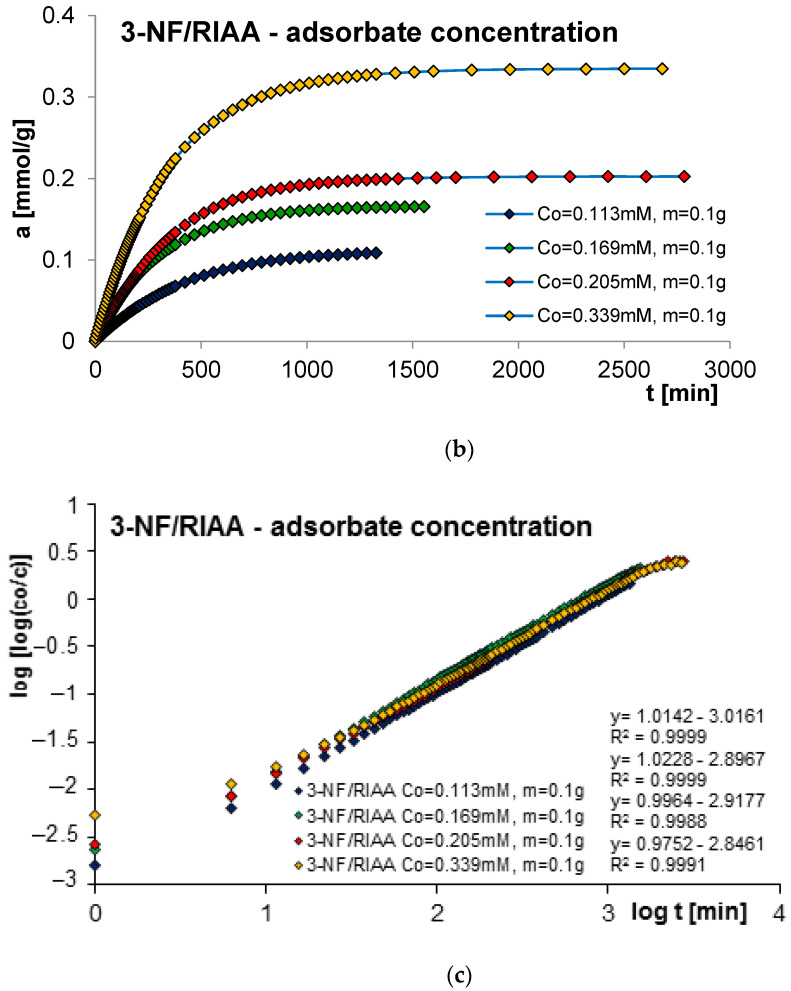
Comparison of the adsorption kinetics of 3-NF on RIAA activated carbon with constant adsorbent mass and variable initial adsorbate concentration presented in the profile of concentration changes over time (**a**), relative adsorption over time (**b**) and in Bangham’s linear coordinates (**c**).

**Figure 13:**
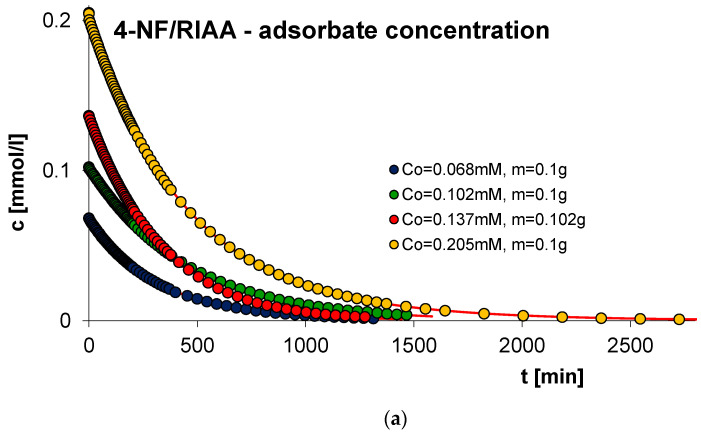

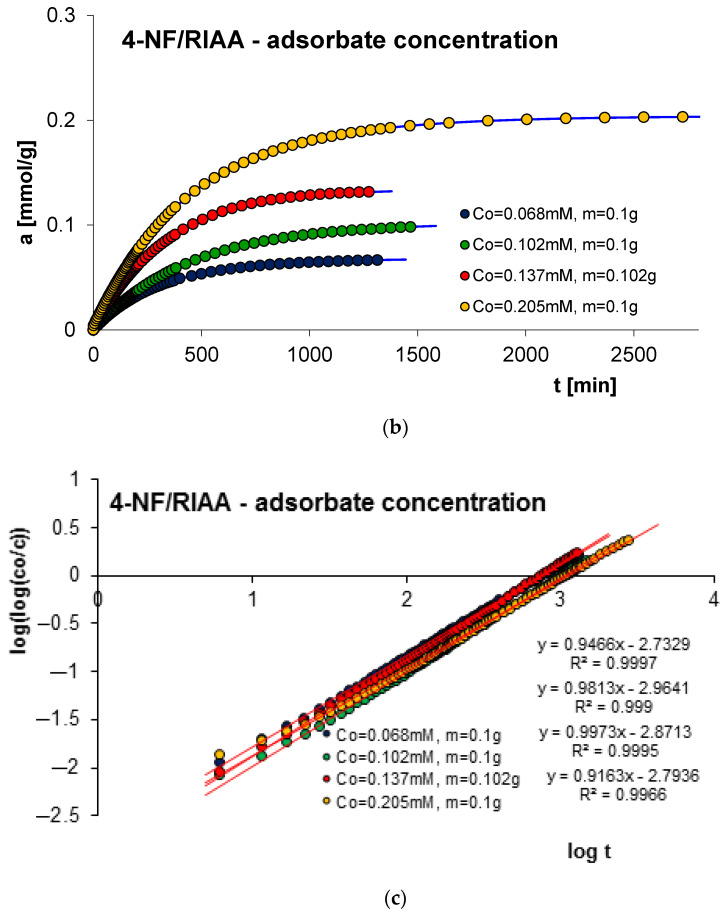
Comparison of the adsorption kinetics of 4-NF on RIAA activated carbon with constant adsorbent mass and variable initial adsorbate concentration presented in the profile of concentration changes over time (**a**), relative adsorption over time (**b**) and in Bangham’s linear coordinates (**c**).

Another factor conditioning the effectiveness of the adsorption process is the concentration of the adsorbate. In order to estimate its impact, the measurements of the kinetics of phenol (F) as well as for 2-, 3- and 4-nitrophenol (2-, 3- and 4-NF) were carried out on RIAA activated carbon. Kinetics measurements were conducted in the following conditions: constant initial adsorbent mass (0.1 g) and variable concentration of the adsorbate (c_0 F_ = 1.4, 0.933, 0.7, 0.467 mM; c_0 2-NF_ = 0.323, 0.205, 0.161, 0.108 mM; c_0 3-NF_ = 0.339, 0.205, 0.169, 0.113 mM; c_0 4-NF_ = 0.205, 0.137, 0.102, 0.068 mM). Figure 10, Figure 11, Figure 12 and Figure 13 present a comparison of the adsorption kinetics of F (Figure 10), 2-NF (Figure 11), 3-NF (Figure 12) and 4-NF (Figure 13) on RIAA shown as the profile of concentration changes over time (Figure 10a, Figure 11a, Figure 12a and Figure 13a), relative adsorption in time (Figure 10b, Figure 11b, Figure 12b and Figure 13b) and in linear Bangham coordinates (Figure 10c, Figure 11c, Figure 12c and Figure 13c).

A clear influence of adsorbate concentration on the process kinetics is observed. As the adsorbate concentration increases, the rate of the adsorption process of F, 2-NF, 3-NF and 4-NF increases. The increase in adsorption kinetics with increasing concentration of the adsorbed substance can be explained as the effect of the concentration difference between the liquid and solid phases. Moreover, the increased driving force overcomes all mass transfer resistances, as a result of which the collision of adsorbate and adsorbent molecules is more likely, which results in a greater binding capacity at higher initial concentrations of the adsorbed substance [50]. Additionally, it was noticed that for all experimental systems, the initial change in concentration over time is proportional to the initial concentration, and the change in relative adsorption is inversely proportional to the initial concentration.

The analysis of the adsorption kinetics of phenol and nitrophenols on RIAA activated carbon depending on the variable adsorbate concentration began with the presentation of experimental data in linear coordinates of Bangham’s relations [49]. It was observed that the graphs are almost linear and their slopes are close to unity (p _F_: 0.70–1.07; p _2-NF_: 0.90–0.97, p _3-NF_: 0.98–1.02; p _4-NF_: 0.92–0.99), which makes it impossible to use the IDM model (*p* = 0.5) to describe the adsorption rate of the tested experimental systems.

Therefore, simple adsorption kinetic equations and models, including FOE, SOE, MOE, f-FOE, f-SOE, f-MOE and m-exp, were applied in the subsequent kinetic data analysis. The obtained relative standard deviations are listed in Table S2. As can be seen, for all experimental systems, the best quality of fit was achieved using the multi-exponential equation (SD(c)/co = 0.109–0.791%), the parameters of which are collected in Table 4.

The analysis of the parameter values included in Table 4 suggests that the adsorption kinetics in the tested experimental systems can be described using one, two or three terms of a multi-exponential equation. However, it should be noted that in the case of systems with three terms, one of them has a very small value. The estimated half-times of adsorption kinetics in the examined experimental systems decrease with increasing adsorbate concentration, which confirms the increase in the adsorption rate in systems with higher availability of organic compounds. The quality of the fit is very good, as confirmed by the low values of the relative standard deviations (SD(c)/c_0 F_: 0.144–0.306%; SD(c)/c_0 2-NF_: 0.211–0.791%; SD(c)/c_0 3-NF_: 0.211–0.792%; SD(c)/c_0 4-NF_: 0.123–0.382%) and the indeterminacy coefficient (1 − R^2^_F_: 1.9 × 10^−5^–9.6 × 10^−5^; 1 − R^2^_2-NF_: 3.9 × 10^−5^–5.0 × 10^−4^; 1 − R^2^_3-NF_: 3.9 × 10^−5^–3.1 × 10^−4^; 1 − R^2^_4-NF_: 1.1 × 10^−5^–1.4 × 10^−4^).

#### 2.2.5. Effect of pH

**Figure 14:**
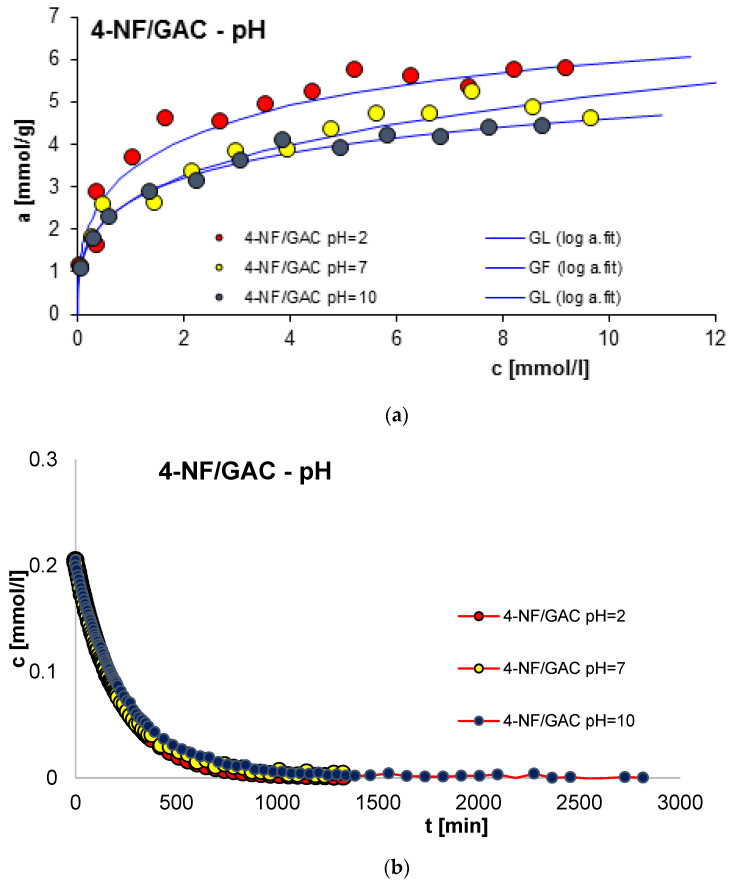

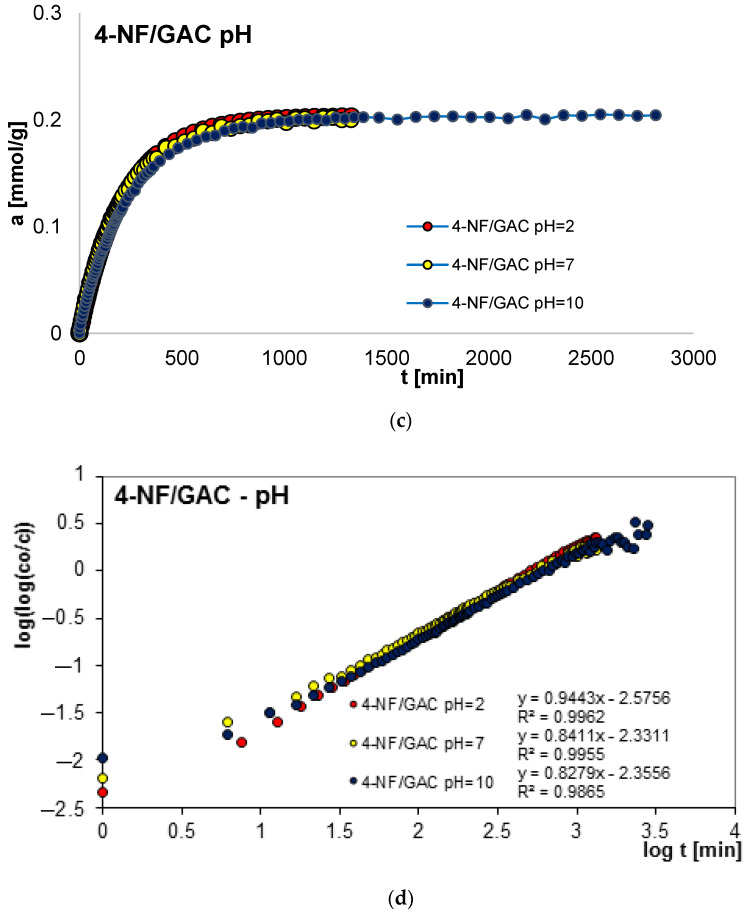
Comparison of adsorption isotherms (**a**) and adsorption kinetics of 4-NF on GAC activated carbon at varying solution pH presented in the profile of concentration changes over time (**b**), relative adsorption over time (**c**) and in linear Bangham coordinates (**d**).

Another very important factor determining the effectiveness of the adsorption process is the solution pH. The study examined the influence of pH on the equilibrium and kinetics of the adsorption of 4-nitrophenol (4-NF) on GAC 1240W (GAC) activated carbon. The tests were carried out at pH 2, 7 and 10. Figure 14 presents a comparison of adsorption isotherms (a) and adsorption kinetic profiles of 4-NF on GAC activated carbon at varying solution pH presented in terms of the dependence of concentration changes over time (b), relative adsorption over time (c) and in linear Bangham coordinates (d).

A clear effect of solution pH on the adsorption efficiency of 4-NF on GAC was observed. A decrease in the rate and amount of adsorption with increasing pH was observed. Under the tested conditions of pH = 2, 7 and 10, the GAC activated carbon has a positive charge [51,52,53]. At the same time, the pKa for 4-NF is 7.1 [54,55], which means that in a strongly acidic environment, almost all para-nitrophenol molecules in the solution are in molecular form [56,57,58]. Therefore, at low pH, electrostatic repulsion almost does not occur; so, the adsorption rate is maximum. The obtained effect may result from the strong chemical bond between the lone pair of electrons of the hydroxyl group of 4-NF and the surface of activated carbon, as well as from the existing forces resulting from dispersion interactions between the aromatic ring of 4-nitrophenol and the graphene surfaces of activated carbon. As the pH increases, in addition to the neutral molecules of 4-NF, p-nitrophenolate anions appear in the system [56,57,58], and at the same time the surface of the adsorbent is still positively charged [51,52,53]. Therefore, it can be expected that the adsorption should be more intensive due to electrostatic attraction. In the case of the experimental systems tested, the opposite effect was obtained. The obtained dependence of adsorption equilibrium and kinetics on pH is probably caused by the presence of OH^-^ ions competing with p-nitrophenolate ions for positively charged adsorption sites of activated carbon.

The adsorption equilibrium experimental data were analyzed by the generalized Langmuir (GL) equation, and its parameters are listed in Table 5. In the case of the 4-NF/GAC system with pH = 7, the heterogeneity parameter n is equal to 1, which causes the GL isotherm to be simplified to the generalized Freundlich (GF) equation. For the remaining measurement series, the parameters n and m are different from 1, so the GL equation is the best for its analysis. The adjusted adsorption capacities range from 4.58 mmol/g to 6.02 mmol/g. These values respond to experimental values. The quality of fit is very good, which is confirmed by high values of determination coefficients (R^2^ = 0.957–0.994) and low values of standard deviations (SD(a) = 0.015–0.050).

Consideration of the obtained kinetic data depending on pH began by presenting them in linear Bangham coordinates [49]. Based on the analysis of the presented relationships, it can be concluded that the graphs are almost linear and their slopes range from 0.84 to 1. This suggests that the adsorption kinetics in the tested systems cannot be described using the IDM model, for which the slope is 0.5.

Considering the above, simple equations and models of adsorption kinetics, including FOE, SOE, MOE, f-FOE, f-SOE, f-MOE and m-exp, were used in the subsequent analysis of the adsorption rate data. The relative standard deviations for studied experimental systems are listed in Table S3. As can be seen, for all experimental systems, the best quality of fit was obtained using the multi-exponential equation (SD(c)/co = 0.095–0.615%), the parameters of which are summarized in Table 6.

Based on the analysis of the data presented in Table 6, it can be concluded that the adsorption of the tested compounds is a complex process, the kinetics of which can be described by two or three terms of the m-exp equation, one of which is small. The quality of the fit is very good, which can be confirmed by the low values of the relative standard deviations SD(c)/co in the range from 0.095% to 0.615% and the low values of the 1 − R^2^ indetermination coefficients in the range of 8.2 × 10^−6^ to 3.6 × 10^−4^.

#### 2.2.6. Effect of the Presence of an Accompanying Substance

**Figure 15:**
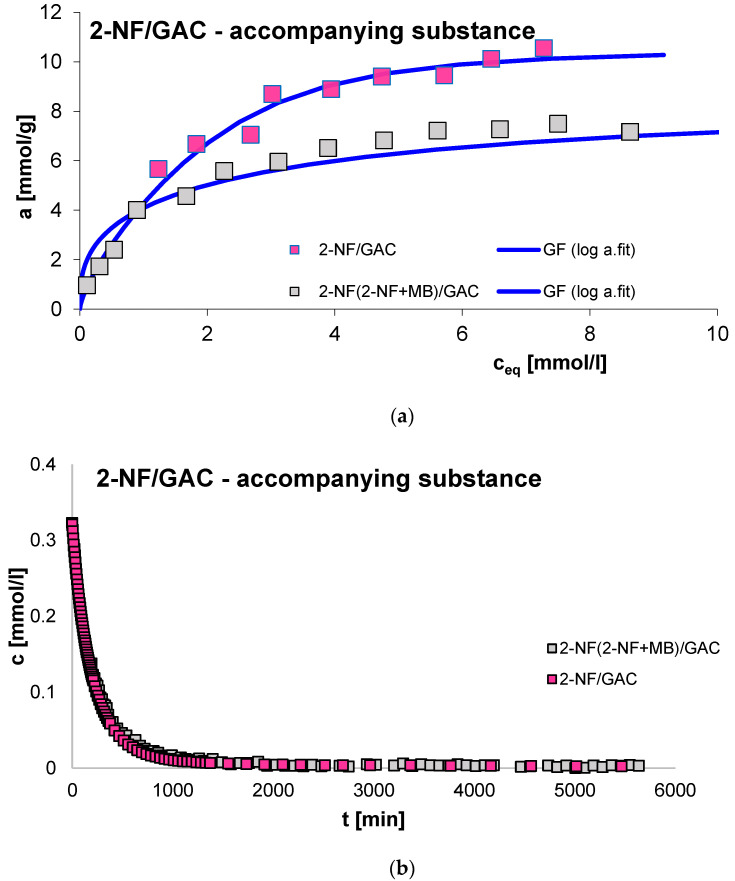

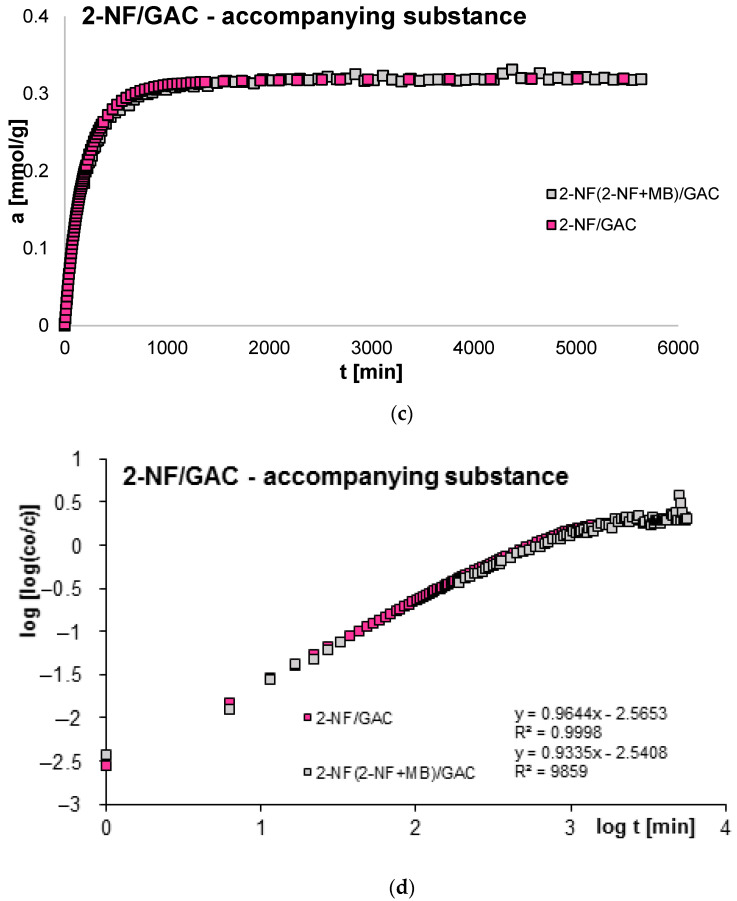
Comparison of adsorption isotherms (**a**) and adsorption kinetics of 2-NF on GAC activated carbon in single- and multi-component systems presented in the profile of concentration changes over time (**b**), relative adsorption over time (**c**) and in linear Bangham coordinates (**d**).

**Figure 16:**
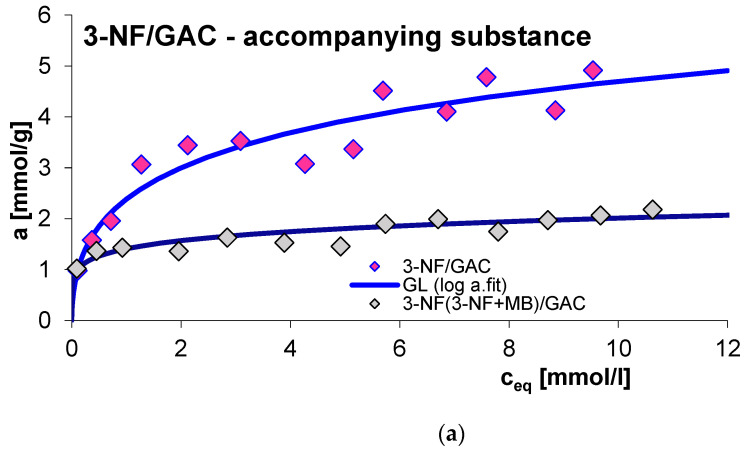

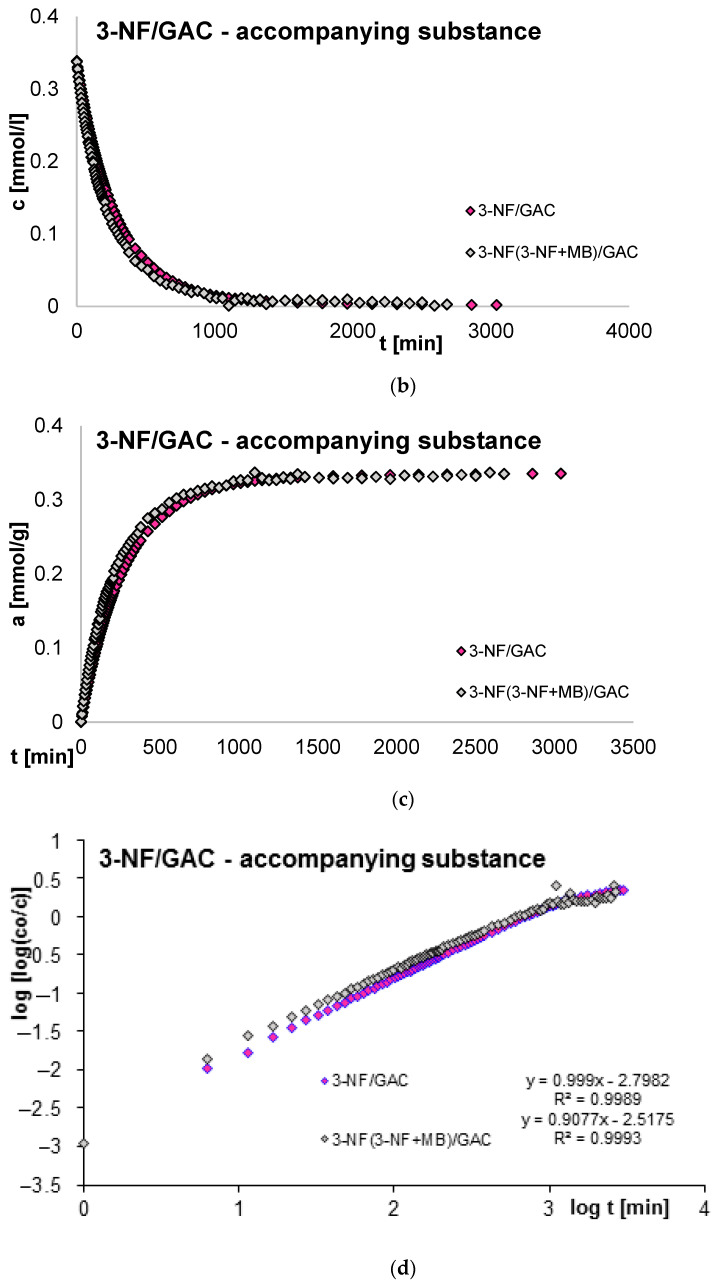
Comparison of adsorption isotherms (**a**) and adsorption kinetics of 3-NF on GAC activated carbon in single- and multi-component systems presented in the profile of concentration changes over time (**b**), relative adsorption over time (**c**) and in linear Bangham coordinates (**d**).

**Figure 17:**
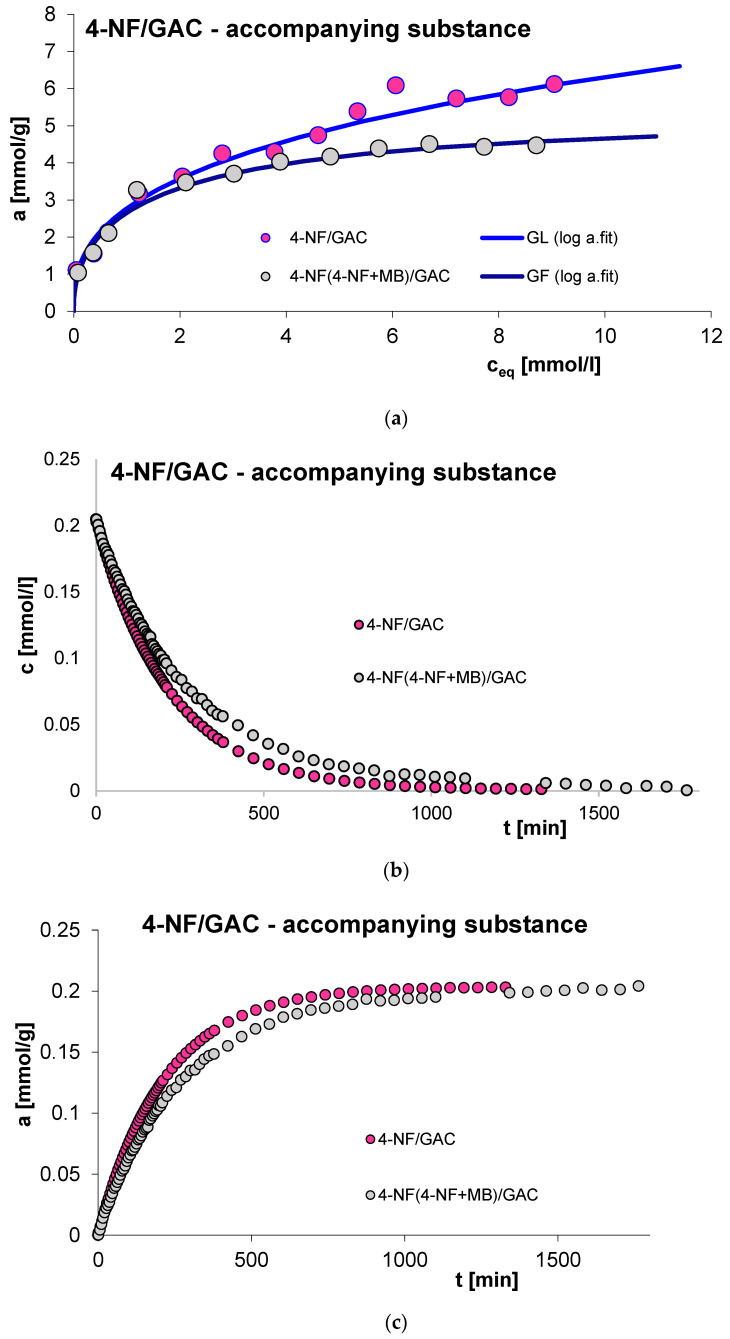

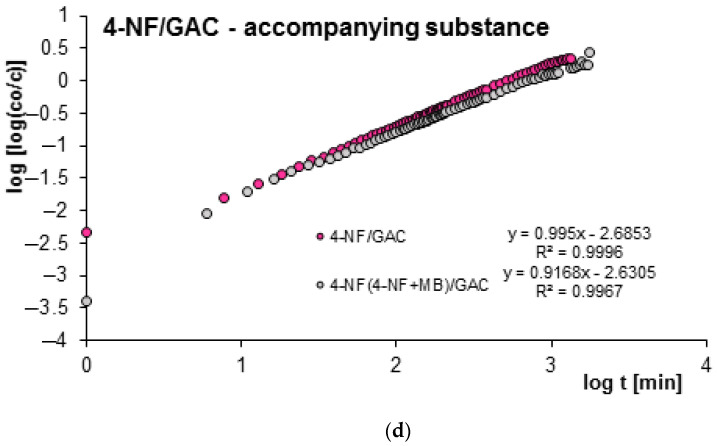
Comparison of adsorption isotherms (**a**) and adsorption kinetics of 4-NF on GAC activated carbon in single- and multi-component systems presented in the profile of concentration changes over time (**b**), relative adsorption over time (**c**) and in linear Bangham coordinates (**d**).

**Figure 18:**
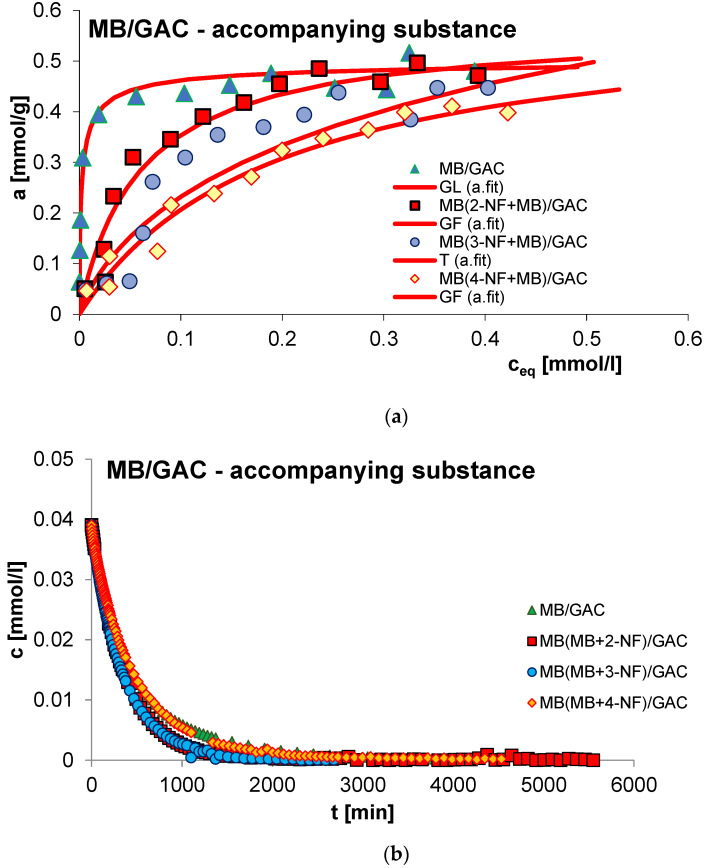

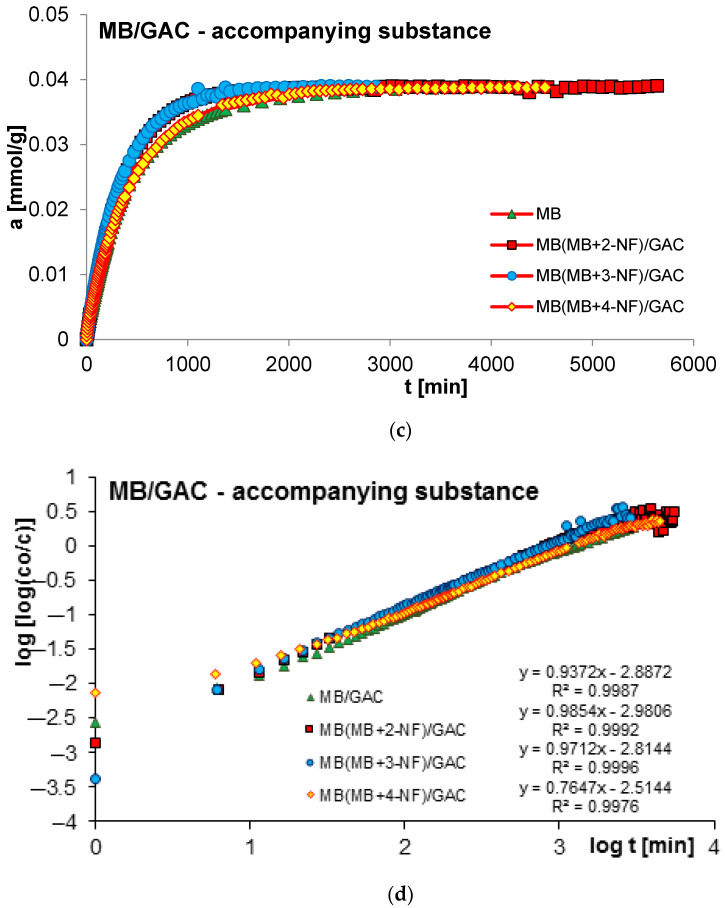
Comparison of adsorption isotherms (**a**) and adsorption kinetics of MB on GAC activated carbon in single- and multi-component systems presented in the profile of concentration changes over time (**b**), relative adsorption over time (**c**) and in linear Bangham coordinates (**d**).

The next factor influencing the adsorption process is the presence of a competing substance. Generally, the rate of the discussed process decreases due to the presence of a co-adsorbate in the experimental system, which is the result of competitive adsorption of solution components. It should also be added that in the case of multi-component systems, smaller adsorbate particles will diffuse more effectively to the adsorbent internal surface. In this study, the influence of the presence of an accompanying substance was investigated for the adsorption of 2-, 3- and 4-nitrophenol (2-, 3- and 4-NF) in systems with methylene blue (MB) on GAC 1240W (GAC) activated carbon. Figure 15, Figure 16, Figure 17 and Figure 18 show a comparison of adsorption isotherms (a) and adsorption kinetics of selected organic compounds on GAC activated carbon in the presence of co-adsorbate presented in the profile of concentration changes over time (b), relative adsorption over time (c) and in linear Bangham coordinates (d).

As can be seen, for all systems the adsorbed amount decreases due to the presence of the accompanying substance. This effect can be explained based on the occurrence of competitive adsorption of solution components in the tested experimental systems [59]. Analogous relationships were noted in the case of adsorption kinetic studies for the sorption of nitrophenols in the presence of methylene blue and the sorption of methylene blue in the presence of 4-nitrophenol. In the case of dye adsorption, an increase in the rate of this process was observed due to the presence of 2- and 3-nitrophenol in the experimental system. 2-NF and 3-NF, unlike MB, have very low solubility in water. Generally, compounds with lower water solubility have a greater affinity for the hydrophobic surface of activated carbon. Additionally, the molecules of 2- and 3-nitrophenol and methylene blue differ significantly in size—the 2-NF and 3-NF molecules are much smaller, which facilitates their diffusion into the microporous spaces of the adsorbent [4]. Moreover, in this part of the study, the concentration of 2- and 3-nitrophenol is much higher than the concentration of methylene blue. Considering the above, in the 2-,(3-)NF+MB/GAC experimental system, 2-,(3-)NF is adsorbed on activated carbon faster, which causes the surface of the used adsorbent to become less hydrophobic, and this, in turn, promotes the adsorption of methylene blue. As a consequence, an increase in the adsorption rate of MB in the presence of 2-NF and 3-NF was observed compared to the adsorption in the MB/GAC system. The above effect was not observed for the adsorption of methylene blue in the presence of 4-nitrophenol, which is probably due to the structure of 4-NF. The arrangement of the substituents in the para position mainly favors penetration into the porous structures of the adsorbent. Therefore, the hydrophobicity of the outer surface of the carbon granules changes to a lesser extent.

The equilibrium data in the various experimental systems were fitted using the generalized Langmuir (GL) isotherm equation, the parameters of which are listed in Table 7. As can be seen, the isotherms for the MB(MB+3-NF)/GAC system can be described by the Tóth (T) equation. Moreover, the full generalized Langmuir isotherm equation can be used to characterize the adsorption of methylene blue and 3- and 4-nitrophenol in individual systems. In other cases, it is best to use the generalized Freundlich (GF) isotherm. The calculated values of adsorption capacities correspond to those determined experimentally. The quality of the fit is very good, as evidenced by the values of the coefficients of determination (R^2^ = 0.932–0.988) and the values of standard deviations (SD(a) = 0.019–0.057).

Consideration of the obtained kinetic data began with presenting them in linear Bangham coordinates [49]. Based on the analysis of the presented relationships, it can be concluded that the graphs are almost linear and their slopes are: 0.93–0.96 for 2-NF, 0.91–1 for 3-NF, 0.92–1 for 4-NF and 0.76–0.99 for MB. This suggests that similarly as in the case of previously analyzed systems, the adsorption kinetics cannot be described using the IDM model, for which the slope is 0.5.

Due to the above, a series of simple equations were used in the further part of the adsorption kinetics analysis, including: FOE, SOE, MOE, f-FOE, f-SOE, f-MOE and m-exp. The obtained values of standard deviations are listed in Table S4. As can be seen, for all the tested systems, the adsorption rate is best characterized by a multi-exponential equation, the adjustment parameters of which are listed in Table 8.

As you can see, the kinetics of methylene blue adsorption in the presence of 2-nitrophenol can be described by one term of the m-exp equation, which is equivalent to first-order kinetics. In other cases, the adsorption rate can be characterized by two or three terms of the m-exp equation, one of which is relatively small. Moreover, a high quality of fit was demonstrated: SD(c)/co in the range from 0.066% to 0.769% and 1 − R^2^ in the range of 3.9 × 10^−6^ to 7.1 × 10^−4^.

## 3. Discussion

The article shows the results of studies on the adsorption of selected aromatic organic compounds on commercially available GAC and RIAA activated carbons. In order to provide detailed and comprehensive information on the adsorption mechanism, the influence of numerous factors on the efficiency of the process of removing organic pollutants from aqueous solutions was determined. The dependence of the adsorption amount on the contact time and the adsorbent grain size for 2-, 3- and 4-nitrophenol on GAC activated carbon was examined. Moreover, the influence of the ratio of the adsorbent mass to the amount of adsorbate on the adsorption kinetics for phenol and its nitro monoderivatives substituted in the ortho, meta and para positions on RIAA activated carbon was estimated. Additionally, the influence of the presence of a competing substance on both the adsorption uptake and rate was examined for methylene blue, 2-nitrophenol, 3-nitrophenol and 4-nitrophenol on GAC activated carbon. The research methodology used enabled strict control of the temperature and the speed of shaking and mixing the adsorption systems. It is also worth noting that the developed adsorption kinetics testing procedure allows for obtaining several dozen (over 80) measurement points. Moreover, detailed processing of the adsorption value and rate data using numerous equations and models of adsorption kinetics and equilibrium enabled the precise determination of the adsorption mechanism in the tested experimental systems. Currently, there are undoubtedly many works in the literature describing the adsorption of aromatic organic compounds on activated carbons. At the same time, there is still a lack of accurate and detailed studies of the equilibrium and kinetics of adsorption, taking into account the influence of numerous factors on this process.

Wasilewska et al. [3] comprehensively investigated the temperature dependencies of the adsorption of 2-, 3- and 4-nitrophenol on GAC activated carbon. The work presents high-quality equilibrium and adsorption kinetics data (the kinetic curves contained even over 100 experimental points) and their detailed analysis. Derylo-Marczewska et al. [4] studied the adsorption of 2-, 3- and 4-nitrophenol on RIAA activated carbon. Adsorption rate tests were carried out at pH = 2.2 and at 25 °C, and high-quality kinetic data were obtained (kinetic curves contained several dozen experimental points). Moreover, the paper presents the temperature dependences of adsorption equilibrium. Yadav et al. [60] studied the adsorption of phenol and p-nitrophenol on magnetic activated carbon synthesized from cauliflower waste. The paper presents the influence of the adsorbent mass and the initial adsorbate concentration on the adsorption kinetics in the experimental systems tested. The kinetic curves contained several experimental points. The influence of solution pH and temperature on the adsorption equilibrium was also presented. Gu et al. [61] studied the adsorption of o-, m- and p-nitrophenol on aminopropyl-modified mesoporous MCM-48. The study examined the influence of solution pH, adsorbent amount and contact time on the efficiency of the adsorption process. Additionally, the adsorption equilibrium data at different temperatures were analyzed. Chaudhary et al. [62] studied the adsorption of phenol, 2-, 3- and 4-nitrophenol on activated carbon developed from demineralized kraft lignin. The paper presents the influence of contact time (several experimental points), initial adsorbent concentration, solution pH and temperature on the adsorption of the tested experimental systems. Magdy et al. [63] studied the adsorption of 2,4-dinitrophenol, 2-nitrophenol and 4-nitrophenol on char ash from animal bones. The influence of contact time (10 experimental points), solution pH and temperature was examined.

## 4. Materials and Methods

### 4.1. Chemicals

The following aromatic organic compounds were used as adsorbates in the research: phenol (F; Sigma-Aldrich, Darmstadt, Germany), 2-nitrophenol (2-NF; Sigma-Aldrich, Beijing, China), 3-nitrophenol (3-NF; Aldrich, St. Quentin Fallavier, France), 4-nitrophenol (4-NF; Fluka, Tokyo, Japan) and methylene blue (MB; Sigma-Aldrich, St. Louis, MO, USA). Their selected physicochemical properties and structures are presented in Table 9.

In this work, adsorption on commercially available activated carbons GAC 1240W (GAC) and RIAA, which were obtained from the company Norit (the Netherlands), was investigated. The textural properties (specific surface area S_BET_, external surface area S_EXT_, total pore volume V_t_, micropore volume V_m_ and average hydraulic pore size d_h_) of these materials were calculated based on the results of nitrogen adsorption/desorption isotherm tests. The values of individual parameters were: S_BET GAC_ = 900 m^2^/g, S_EXT GAC_ = 523 m^2^/g, V_t GAC_ = 0.52 cm^3^/g, V_mic GAC_ = 0.20 cm^3^/g, d_h GAC_ = 2.31 nm [3]; S_BET RIAA_ = 1390 m^2^/g, S_EXT RIAA_ = 48 m^2^/g, V_t RIAA_ = 0.67 cm^3^/g, V_mic RIAA_ = 0.31 cm^3^/g, d_h RIAA_ = 1.93 nm [4].

### 4.2. Methods

#### 4.2.1. Potentiometric Titration

Potentiometric titration measurements were performed using a set consisting of a Dosimat 765 automatic burette (Metrohm, Zofingen, Switzerland), a PHM 240 pH meter with a pHC2401 combined electrode (Radiometer, Neuilly-Plaisance, France), an Ecoline RE 207 thermostat (Lauda, Germany), a thermostatted measuring vessel and a control computer with our own software (“Titr_v3” program written by W. Janusz and A.W. Marczewski, Faculty of Chemistry, MCSU in Lublin). These studies allowed for the determination of surface charge density and zero charge points for GAC and RIAA activated carbons. For this purpose, 30 cm^3^ of 0.1 mol/L NaCl was poured into a measuring vessel with a thermometer, an electrode and a mechanical stirrer. Then, after reaching equilibrium, the solution was acidified (0.3 cm^3^ HCl; 0.2 mol/L) and a prepared portion of carbon (0.1 g) was added. The suspension was titrated with 0.1 mol/L NaOH while monitoring pH changes. The tests were carried out at 25 °C.

#### 4.2.2. TEM

A Titan G2 60-300 transmission electron microscope (FEI, Hillsboro, OR, USA) was used to perform TEM micrographs. For this purpose, a small amount of carbon was crushed in a mortar, added to alcohol and exposed to ultrasound. In the next stage, the sample was placed on a copper mesh, which was placed in the measuring chamber. The structure of selected activated carbons was examined using the bright field technique in the TEM mode with chromatic aberration correction, under conditions of an electron beam accelerating voltage of 300 kV.

#### 4.2.3. SEM

A Quanta 3D FEG scanning electron–ion microscope (FEI, USA) was used to make SEM micrographs. GAC and RIAA activated carbon samples were introduced on an aluminum table with self-adhesive carbon foil. Observations were carried out in high-vacuum conditions with an accelerating voltage of 5 kV.

#### 4.2.4. Adsorption Equilibrium

The influence of several factors on the adsorption equilibrium on GAC activated carbon was investigated. The influence of the adsorbent grain size and contact time was determined for adsorption of 2-nitrophenol, 3-nitrophenol and 4-nitrophenol. The dependence of sorption efficiency on pH was examined for the system with 4-nitrophenol. The influence of the presence of an accompanying substance for the systems containing 2-, 3- or 4-NF and methylene blue was also determined. Adsorption equilibrium measurements were performed in Erlenmeyer flasks. The volumes of organic compound solutions were 100 cm^3^ and their pH was equal to 2 (but for the series on the impact of pH, its value was established at 2, 7 and 10). In the tests, the GAC carbon fraction with a grain size >0.5 mm was used (but for the series examining the influence of adsorbent grain size, the fractions with a diameter of <0.3 mm, 0.3–0.5 mm and >0.5 mm were used), and the adsorbent weights were equal to 0.05 g. The systems prepared in this way were shaken for 7 days (but for the series examining the influence of contact time, the adsorption process lasted 1, 4 or 7 days) at a temperature 25 °C with mixing speed of 110 rpm. Then, the solutions were decanted and their absorbance was determined using a Cary 4000 UV–Vis spectrophotometer (Varian, Belrose, Australia). All spectrophotometric measurements were carried out with the registration of spectra in the range of 200–800 nm. Peak maximum occurred at wavelengths of 278 nm, 273 nm, 317 nm and 664 nm for 2-NF, 3-NF, 4-NF and MB, respectively.

The Marczewski–Jaroniec isotherm (M-J; also known as the generalized Langmuir (GL) isotherm) was used to analyze the equilibrium data. This equation describes adsorption in a liquid/solid system well, which is characterized by energy heterogeneity. The M-J isotherm has the following form:

(1) $$\theta ={\left[\frac{{\left(\bar{K}\cdot c_{eq}\right)}^{n}}{{1+\left(\bar{K}\cdot c_{eq}\right)}^{n}}\right]}^{\frac{m}{n}}$$

where: θ—surface coverage, θ = a/a_m_, m, n—heterogeneity parameters, K—the adsorption equilibrium constant. In particular cases, GL transforms into the following isotherms: the generalized Freundlich (GF; n = 1), Langmuir–Freundlich (LF; m = n), Tóth (T; m = 1) or Langmuir (L; m = n = 1) [64,65].

#### 4.2.5. Adsorption Kinetics

The influence of several factors on the adsorption kinetics on GAC and RIAA activated carbons was investigated. The effects of adsorbent mass and adsorbate concentration were investigated for the adsorption of phenol, 2-nitrophenol, 3-nitrophenol and 4-nitrophenol on RIAA. The adsorption rate depending on the solution pH was examined for systems with 4-nitrophenol on GAC. However, the influence of the presence of an accompanying substance on the adsorption kinetics was examined for methylene blue and 2-, 3- or 4-nitrophenol. Adsorption kinetic studies were carried out in a thermostatted vessel. The ratio of the adsorbent mass to the volume of the adsorbate solution was 1 g/L (but for the series regarding the study of the effect of adsorbent mass, the weights were 0.05 g, 0.1 g, 0.15 g and 0.2 g). The adsorption process was carried out at a pH = 2 (but for the series on the impact of pH, its value was 2, 7 and 10) at a temperature 25 °C. The systems were mixed using a mechanical mixer with digital control of the mixing speed, which was 110 rpm. Changes in concentration over time were measured using the spectrometric method (Cary 100 UV–Vis spectrophotometer; Varian, Australia). All spectrophotometric investigations were performed with the registration of spectra in the range from 200 to 800 nm. Peak maximum occurred at wavelengths of 270 nm, 278 nm, 273 nm, 317 nm and 664 nm for F, 2-NF, 3-NF, 4-NF and MB, respectively.

The adsorption rate data were analyzed using the following kinetic equations: first order (FOE), second order (SOE), mixed order (MOE) and their fractal equivalents (f-FOE, f-SOE, f-MOE) as well as a multi-exponential (m-exp) equation.

The first-order kinetic equation (FOE)/the pseudo-first-order equation (PFOE) has the following form:

(2) $$ln\left(c_{eq}-c\right)=ln{(c}_{eq}-c_{o})-k_{1}$$

or

(3) $$ln\left(a_{eq}-a\right)=lna_{eq}-k_{1}t$$

where: c—the temporary concentration, a—the actual adsorbed amount, the “o” and “eq” indices relate to the initial and equilibrium values, k_1_—the adsorption rate coefficient [66,67].

The second-order equation (SOE)/the pseudo-second-order equation (PSOE) takes the following form:

(4) $$a=a_{eq}\left[k_{2}t/\left(1+k_{2}t\right)\right]$$

or its linear forms:

(5) $$t/a=\left(1/a_{eq}\right)\left(1/k_{2}+t\right)$$

and

(6) $$a=a_{eq}-\left(1/k_{2}\right)\left(a/t\right)$$

where: k_2_ = k_2a_a_eq_ and k_2a_ are the rate coefficients for pseudo-second-order kinetics [67,68,69].

The mixed 1,2-order kinetic equation (MOE) is a generalization of the first- and second-order kinetics. MOE can be expressed as a relative adsorption process, F, over time:

(7) $$F=a/a_{eq}=\frac{1-exp\left(-k_{1}t\right)}{1-f_{2}exp\left(-k_{1}t\right)}$$

or

(8) $$ln\left(\frac{1-F}{1-f_{2}F}\right)=-k_{1}t$$

where f_2_ < 1—the normalized share of the second-order process in the kinetics [70,71].

The fractal-like MOE equation (f-MOE) takes into account the nonideality effects. It takes the following form:

(9) $$F=\frac{{1-exp\left(-k_{1}t\right)}^{p}}{{1-f_{2}exp\left(-k_{1}t\right)}^{p}}$$

where: p—the fractal coefficient. In particular cases f-MOE can transform to the following equations: MOE (p = 0), f-FOE (f_2_ = 0) and f-SOE (f_2_ = 1) [72,73]

The multi-exponential equation (m-exp) is often applied for adsorption on heterogeneous solids. The m-exp can characterize the series of the first-order processes or the follow-up processes. It has the following form:

(10) $$c=\left(c_{0}-c_{eq}\right)\sum_{i=1}^{n}f_{i}exp\left(-k_{i}t\right)+c_{eq}$$

or

(11) $$c=c_{0}-c_{0}u_{eq}\sum_{i=1}^{n}f_{i}\left[1-exp\left(-k_{i}t\right)\right]$$

where: “i”—the term of the m-exp equation, k_i_—the rate coefficient, u_eq_ = 1 − c_eq_/c_0_—the relative loss of adsorbate from the solution [52].

## 5. Conclusions

The influence of numerous factors on the adsorption efficiency of selected organic compounds was investigated. The adsorption of phenol (F), 2-nitrophenol (2-NF), 3-nitrophenol (3-NF), 4-nitrophenol (4-NF) and methylene blue (MB) on commercially available activated carbons GAC 1240W (GAC) and RIAA was tested. The dependence of the adsorption equilibrium on the adsorbent grain size (2-, 3-, 4-NF/GAC) and contact time (2-, 3-, 4-NF/GAC) was examined. Adsorption kinetics was studied depending on the adsorbent mass (F, 2-, 3-, 4-NF/RIAA) and adsorbate concentration (F, 2-, 3-, 4-NF/RIAA). The influence of solution pH (4-NF/GAC) and the presence of an accompanying substance (2-, 3-, 4-NF+MB/GAC) on both the rate and adsorption value was also examined.

In the tested experimental systems, a clear influence of the above-mentioned factors on the efficiency of removing chosen organic pollutants from aqueous solutions was observed. It was reported that a longer contact time and the use of smaller adsorbent grain fractions result in an increase in the adsorption value. It was noted that depending on the contact time (1 day, 4 and 7 days), the adsorbed amount varied from 4.32–10.55 mmol/g, 1.93–4.91 mmol/g and 3.62–6.13 mmol/g for 2-NF, 3-NF and 4-NF, respectively. Longer contact of nitrophenols with GAC activated carbon enabled sorption not only on its surface but also penetration into its porous structure. Therefore, all experimental systems were shaken for 7 days. Moreover, it was observed that the adsorption amount depending on the adsorbent grain size (<0.3 mm, 0.3–0.5 mm and >0.5 mm) varies within the range of 10.55–11.28 mmol/g, 4.91–5.94 mmol/g and 6.13–7.0 mmol/ g for 2-NF, 3-NF and 4-NF, respectively. The highest adsorbed amounts were recorded in systems with activated carbon of the smallest grain size. At the same time, when working with dusty materials, there are problems with their complete recovery for reuse. Therefore, GAC activated carbon with a grain diameter >0.5 mm was used for all studies.

Additionally, it was shown that adsorption kinetics is higher in systems with higher adsorbent mass and adsorbate concentration. It was shown that, depending on the adsorbent weight used, the half-times ranged from 222.4–655.4 min, 117.2–340.6 min, 117.7–457.0 min and 154.2–553.5 min for the adsorption of F, 2-NF, 3-NF and 4-NF on RIAA carbon. As expected, the fastest adsorption was observed in the systems for which the highest mass of adsorbent was used. At the same time, in order to maintain economy and not to lose the effectiveness of the sorption process, it was determined that the optimal ratio of carbon mass to solution volume should be 1 g/L.

In addition, it was also shown that for the adsorption of 4-nitrophenol on GAC carbon, a higher rate and adsorbed amount were observed at lower pH values. Moreover, it was shown that, depending on the pH of the environment (2, 7 and 10), the adsorbed amounts of 4-NF ranged from 4.43 to 5.82 mmol/g. In an acidic environment, 4-nitrophenol molecules exist in molecular form, as a result of which the problem of competitive adsorption for the active sites of the adsorbent between hydroxyl and p-nitrophenolate ions disappears. Therefore, all tests were carried out at pH = 2.

It was also shown that in the tested experimental systems the adsorption efficiency is limited due to the presence of the accompanying substance. It was reported that the adsorption rate in multi-component systems, compared to single-component systems, is much lower and amounts reduced to 7.17 mmol/g, 2.18 mmol/g and 4.47 mmol/g for the sorption of 2-NF, 3-NF and 4-NF in the presence of MB, respectively, were observed. Additionally, it was found that the adsorption rate of methylene blue was reduced from 0.52 mmol/g to even 0.40 mmol/g. It was also shown that the adsorption kinetics of nitrophenols in the presence of co-adsorbate was lower. Interestingly, in the case of methylene blue sorption, the presence of 2- and 3-nitrophenol promoted the adsorption rate.

The isotherm of the generalized Langmuir equation (R^2^ = 0.912–0.996) was used to analyze the equilibrium data. For many experimental systems tested, the adsorption process is perfectly described by the full form of this equation. At the same time, in some experiments one of the heterogeneity parameters (m or n) is equal to 1, as a result of which the GL equation is transformed into the T and GF isotherms. From a practical point of view, this indicates the occurrence of medium heterogeneity effects in the tested experimental systems.

The initial analysis of the kinetic data using Bangham’s linear relations, in the case of all experimental systems tested, excluded the possibility of using the diffusion model. Due to the above, simple equations FOE, SOE, MOE, f-FOE, f-SOE, f-MOE and m-exp were used to analyze the adsorption kinetics. For all experimental systems tested, the adsorption rate was best described by the multi-exponential equation (1 − R^2^ = (1.0 × 10^−6^)–(8.2 × 10^−4^)).

## Figures and Tables

**Table 1:** Parameters of the generalized Langmuir eq. describing adsorption of 2-, 3- and 4-nitrophenol from dilute aqueous solutions on GAC activated carbon with different grain sizes: <0.3 mm, 0.3–0.5 mm and >0.5 mm.

System	Isotherm Type	a_m_^a^	m ^b^	n ^b^	logK ^c^	R^2 d^	SD(a) ^e^
2-NF/GAC < 0.3 mm	GF	11.46	0.93	1	−0.07	0.996	0.022
2-NF/GAC 0.3–0.5 mm	GF	10.87	0.82	1	−0.40	0.993	0.028
2-NF/GAC > 0.5 mm	GF	10.71	0.39	1	−0.54	0.988	0.019
3-NF/GAC < 0.3 mm	GF	6.02	0.41	1	−0.84	0.987	0.025
3-NF/GAC 0.3–0.5 mm	GF	5.32	0.51	1	−0.78	0.987	0.026
3-NF/GAC > 0.5 mm	GL	5.13	0.30	0.91	−0.59	0.964	0.051
4-NF/GAC < 0.3 mm	GF	7.11	0.69	1	−0.27	0.987	0.028
4-NF/GAC 0.3–0.5 mm	GF	6.79	0.47	1	−0.76	0.954	0.053
4-NF/GAC > 0.5 mm	GL	6.02	0.27	0.84	−0.12	0.973	0.050

^a^ a_m_—adsorption capacity, ^b^ m and n—parameters of energetic heterogeneity, ^c^ logK—logarithm of the adsorption equilibrium constant, ^d^ R^2^—determination coefficient, ^e^ SD(a)—standard deviation.

**Table 2:** Parameters of the generalized Langmuir eq. describing adsorption of 2-, 3- and 4-nitrophenol from dilute aqueous solutions on GAC activated carbon with different contact times: 1 day, 4 days and 7 days.

System	Isotherm Type	a_m_^a^	m ^b^	n ^b^	logK ^c^	R^2 d^	SD(a) ^e^
2-NF/GAC 1 day	GF	4.33	0.95	1	−0.27	0.972	0.046
2-NF/GAC 4 days	GL	6.51	0.63	0.61	−0.79	0.943	0.069
2-NF/GAC 7 days	GF	10.71	0.39	1	−0.54	0.988	0.019
3-NF/GAC 1 day	GF	2.01	0.69	1	−0.57	0.961	0.039
3-NF/GAC 4 days	GL	2.91	0.45	0.19	−0.44	0.912	0.054
3-NF/GAC 7 days	GL	5.13	0.30	1	−0.59	0.964	0.051
4-NF/GAC 1 day	T	3.83	1	0.43	−0.66	0.947	0.071
4-NF/GAC 4 days	GL	5.19	0.53	0.63	−1.36	0.991	0.023
4-NF/GAC 7 days	GL	6.02	0.27	0.84	−0.12	0.973	0.050

^a^ a_m_—adsorption capacity, ^b^ m and n—parameters of energetic heterogeneity, ^c^ logK—logarithm of the adsorption equilibrium constant, ^d^ R^2^—determination coefficient, ^e^ SD(a)—standard deviation.

**Table 3:** The optimized parameters of multi-exponential eq. for F, 2-, 3- and 4-NF adsorption kinetics on RIAA activated carbon (constant initial concentration and variable adsorbent mass).

System	f_1_^a^, log k_1_^b^	f_2_^a^, log k_2_^b^	f_3_^a^, log k_3_^b^	u_eq_^c^	t_1/2_^d^ [min]	SD(c)/c_o_^e^ [%]	1 − R^2 f^
F/RIAA Co = 1.4 mM, m = 0.05 g	0.598; −2.58	0.402; −4.48	-	0.902	655.4	0.163	5.1 × 10^−5^
F/RIAA Co = 1.4 mM, m = 0.1 g	0.562; −2.45	0.344; −3.06	0.094; −3.94	0.896	358.9	0.190	3.7 × 10^−5^
F/RIAA Co = 1.4 mM, m = 0.15 g	0.753; −2.48	0.247; −3.3	-	1	291.1	0.832	8.2 × 10^−4^
F/RIAA Co = 1.4 mM, m = 0.2 g	0.008; 1.30	0.733; −2.38	0.260; 2.94	0.952	222.4	0.094	8.5 × 10^−6^
2-NF/RIAA Co = 0.323 mM, m = 0.05 g	0.285; −2.08	0.715; −2.94	-	0.998	340.6	0.512	2.6 × 10^−4^
2-NF/RIAA Co = 0.323 mM, m = 0.1 g	0.403; −2.15	0.597; −2.50	-	0.997	156.8	0.344	1.1 × 10^−4^
2-NF/RIAA Co = 0.323 mM, m = 0.15 g	0.581; −2.28	0.419; −2.52	-	0.999	144.0	0.251	5.6 × 10^−5^
2-NF/RIAA Co = 0.323 mM, m = 0.2 g	0.197; −1.95	0.803; −2.30	-	0.995	117.2	0.455	2.0 × 10^−4^
3-NF/RIAA Co = 0.339 mM, m = 0.05 g	0.863; −2.78	0.137; −3.08	-	0.991	457.0	0.246	4.5 × 10^−5^
3-NF/RIAA Co = 0.339 mM, m = 0.1 g	1.000; −2.54	-	-	0.998	240.0	0.231	4.5 × 10^−5^
3-NF/RIAA Co = 0.339 mM, m = 0.15 g	1.000; −2.42	-	-	1.000	183.1	0.480	1.9 × 10^−4^
3-NF/RIAA Co = 0.339 mM, m = 0.2 g	0.197; −1.95	0.803; −2.30	-	0.995	117.7	0.451	2.0 × 10^−4^
4-NF/RIAA Co = 0.205 mM, m = 0.05 g	0.005; 1.78	0.494; −2.78	0.501; −3.04	1	553.5	0.086	7.7 ×·10^−6^
4-NF/RIAA Co = 0.205 mM, m = 0.1 g	1.000; −2.65	-	-	1	309.3	0.175	3.0 ×·10^−5^
4-NF/RIAA Co = 0.205 mM, m = 0.15 g	0.867; −2.36	0.133; −2.67	-	1	171.0	0.327	9.9·× 10^−5^
4-NF/RIAA Co = 0.205 mM, m = 0.2 g	0.003; 1.51	0.997; −2.35	-	0.997	154.2	0.106	1.0·× 10^−5^

^a^ f_1_, f_2_, f_3_—the terms of m-exp equation, ^b^ log k_1_, log k_2_, log k_3_—logarithm of the rate constant, ^c^ u_eq_—the relative loss of adsorbate from the solution, ^d^ t_1/2_—half-time, ^e^ SD(c)/c_o_—relative standard deviation, ^f^ 1 − R^2^—indetermination coefficient.

**Table 4:** The optimized parameters of multi-exponential eq. for F, 2-, 3- and 4-NF adsorption kinetics on RIAA activated carbon (constant adsorbent mass and variable initial concentration).

System	f_1_^a^, log k_1_^b^	f_2_^a^, log k_2_^b^	f_3_^a^, log k_3_^b^	u_eq_^c^	t_1/2_^d^ [min]	SD(c)/c_o_^e^ [%]	1 − R^2 f^
F/RIAA Co = 1.4 mM, m = 0.1 g	0.562; −2.45	0.344; −3.06	0.094; −3.94	0.896	358.9	0.190	3.7 × 10^−5^
F/RIAA Co = 0.933 mM, m = 0.1 g	0.08; 0.54	0.674; −2.42	0.246; −2.96	0.924	207.3	0.306	9.6 × 10^−5^
F/RIAA Co = 0.7 mM, m = 0.1 g	0.667; −2.55	0.256; −3.09	0.077; −4.71	1	371.5	0.144	1.91 × 10^−5^
F/RIAA Co = 467 mM, m = 0.1 g	0.870; −2.60	0.13; −3.24	-	0.951	319.5	0.239	4.8 × 10^−5^
2-NF/RIAA Co = 0.323 mM, m = 0.1 g	0.403; −2.15	0.597; −2.50	-	0.997	156.8	0.344	1.1 × 10^−4^
2-NF/RIAA Co = 0.205 mM, m = 0.1 g	1.000; −2.55	-	-	1	245.3	0.791	5.0 × 10^−4^
2-NF/RIAA Co = 0.161 mM, m = 0.1 g	1.000; −2.48	-	-	0.999	208.5	0.211	3.9 × 10^−5^
2-NF/RIAA Co = 0.108 mM, m = 0.1 g	1.000; −2.48	-	-	1	281.6	0.556	3.1 × 10^−4^
3-NF/RIAA Co = 0.339 mM, m = 0.1 g	0.331; −2.10	0.669; −2.58	-	1	177.9	0.252	6.8 × 10^−5^
3-NF/RIAA Co = 0.205 mM, m = 0.1 g	1.000; −2.55	-	-	1	247.3	0.792	5.1 × 10^−4^
3-NF/RIAA Co = 0.169 mM, m = 0.1 g	1.000; −2.48	-	-	0.999	208.5	0.211	3.9 × 10^−5^
3-NF/RIAA Co = 0.113 mM, m = 0.1 g	1.000; −2.61	-	-	1	281.6	0.556	3.1 × 10^−4^
4-NF/RIAA Co = 0.205 mM, m = 0.1 g	0.016; 1.36	0.839; −2.62	0.145; −2.88	1	307.1	0.123	1.3 × 10^−5^
4-NF/RIAA Co = 0.137 mM, m = 0.1 g	1.000; −2.52	-	-	1	229.9	0.382	1.4 × 10^−4^
4-NF/RIAA Co = 0.102 mM, m = 0.1 g	1.000; −2.65	-	-	1	309.3	0.175	3.0 × 10^−5^
4-NF/RIAA Co = 0.068 mM, m = 0.1 g	0.007; −0.30	0.447; −2.37	0.547; −2.61	1	215.6	0.109	1.1 × 10^−5^

^a^ f_1_, f_2_, f_3_—the terms of m-exp equation, ^b^ log k_1_, log k_2_, log k_3_—logarithm of the rate constant, ^c^ u_eq_—the relative loss of adsorbate from the solution, ^d^ t_1/2_—half-time, ^e^ SD(c)/c_o_—relative standard deviation, ^f^ 1 − R^2^—indetermination coefficient.

**Table 5:** Parameters of the generalized Langmuir eq. describing adsorption of 4-nitrophenol from dilute aqueous solutions on GAC activated carbon at varying pH: 2, 7 and 10.

System	Isotherm Type	a_m_^a^	m ^b^	n ^b^	logK ^c^	R^2 d^	SD(a) ^e^
4-NF/GAC pH = 2	GL	6.02	0.27	0.84	−0.12	0.973	0.050
4-NF/GAC pH = 7	GF	4.97	0.29	1	−3.04	0.957	0.041
4-NF/GAC pH = 10	GL	4.58	0.37	0.49	−1.06	0.994	0.015

^a^ a_m_—sorption capacity, ^b^ m and n—parameters of heterogeneity, ^c^ logK—logarithm of the adsorption equilibrium constant, ^d^ R^2^—determination coefficient, ^e^ SD(a)—standard deviation.

**Table 6:** The optimized parameters of multi-exponential eq. for 4-NF adsorption on GAC activated carbon at varying pH: 2, 7 and 10.

System	f_1_^a^, log k_1_^b^	f_2_^a^, log k_2_^b^	f_3_^a^, log k_3_^b^	u_eq_^c^	t_1/2_^d^ [min]	SD(c)/c_o_^e^ [%]	1 *−* R^2 f^
4-NF/GAC pH = 2	0.010; −1.64	0.990; −2.34	-	0.997	204.6	0.095	8.2 × 10^−6^
4-NF/GAC pH = 7	0.026; −0.25	0.435; −2.16	0.539; −2.47	0.991	141.0	0.615	3.6 × 10^−4^
4-NF/GAC pH = 10	0.024; −1.78	0.875; −2.36	0.101; −2.75	0.997	167.0	0.434	1.6 × 10^−4^

^a^ f_1_, f_2_, f_3_—the terms of m-exp equation, ^b^ log k_1_, log k_2_, log k_3_—logarithm of the rate constant, ^c^ u_eq_—the relative loss of adsorbate from the solution, ^d^ t_1/2_—half-time, ^e^ SD(c)/c_o_—relative standard deviation, ^f^ 1 − R^2^—indetermination coefficient.

**Table 7:** Parameters of the generalized Langmuir eq. describing adsorption of MB, 2-, 3- and 4-nitrophenol from dilute aqueous solutions on GAC activated carbon in single- and multi-component systems: 2-NF+MB, 3-NF+MB and 4-NF+MB.

System	Isotherm Type	a_m_^a^	m ^b^	n ^b^	logK ^c^	R^2 d^	SD(a) ^e^
2-NF/GAC	GF	10.71	0.39	1	−0.54	0.988	0.019
2-NF(2-NF+MB)/GAC	GF	3.47	0.34	1	−0.32	0.958	0.047
3-NF/GAC	GL	5.13	0.30	0.96	−0.59	0.964	0.051
3-NF(3-NF+MB)/GAC	GF	2.25	0.15	1	−7.44	0.953	0.057
4-NF/GAC	GL	6.02	0.27	0.84	−0.12	0.973	0.050
4-NF(4-NF+MB)/GAC	GF	4.52	0.44	1	−0.62	0.980	0.030
MB/GAC	GL	0.50	0.61	0.63	2.62	0.981	0.026
MB(MB+2-NF)/GAC	GF	0.48	0.95	1	1.19	0.947	0.044
MB(MB+3-NF)/GAC	T	0.45	1	0.47	0.76	0.932	0.049
MB(MB+4-NF)/GAC	GF	0.41	0.96	1	0.71	0.971	0.024

^a^ a_m_—sorption capacity, ^b^ m and n—parameters of heterogeneity, ^c^ logK—logarithm of the adsorption equilibrium constant, ^d^ R^2^—determination coefficient, ^e^ SD(a)—standard deviation.

**Table 8:** The optimized parameters of multi-exponential eq. for MB, 2-, 3- and 4-nitrophenol adsorption from dilute aqueous solutions on GAC activated carbon in single- and multi-component systems: 2-NF+MB, 3-NF+MB and 4-NF+MB.

System	f_1_^a^, log k_1_^b^	f_2_^a^, log k_2_^b^	f_3_^a^, log k_3_^b^	u_eq_^c^	t_1/2_^d^ [min]	SD(c)/c_o_^e^ [%]	1 − R^2 f^
2-NF/GAC	0.767; −2.06	0.233; −2.50	-	0.992	133.5	0.066	3.9 × 10^−6^
2-NF(2-NF+MB)/GAC	0.753; −2.30	0.164; −2.83	0.083; −1.74	0.995	145.3	0.769	6.4 × 10^−4^
3-NF/GAC	0.067; −2.04	0.913; −2.47	0.020; −3.43	1	157.1	0.116	1.1 × 10^−5^
3-NF(3-NF+MB)/GAC	0.636; −2.21	0.364; −2.67	-	1	196.3	0.883	7.1 × 10*^−4^*
4-NF/GAC	0.010; −1.64	0.990; −2.34	-	0.997	193.2	0.095	8.2 × 10^−6^
4-NF(4-NF+MB)/GAC	0.832; −2.38	0.168; −2.82	-	1	204.6	0.528	2.6 × 10^−4^
MB/GAC	0.600; −2.42	0.400; −3.01	-	1	290	0.513	1.0 × 10^−6^
MB(MB+2-NF)/GAC	1.000; −2.56	-	-	0.995	250.4	0.604	3.6 × 10^−4^
MB(MB+3-NF)/GAC	0.072; −2.01	0.020; −2.61	0.908; −2.57	1	234.5	0.506	2.0 × 10^−4^
MB(MB+4-NF)/GAC	0.024; −0.03	0.426; −2.50	0.550; −2.81	0.994	307.9	0.227	3.8 × 10^−5^

^a^ f_1_, f_2_, f_3_—the terms of m-exp equation, ^b^ log k_1_, log k_2_, log k_3_—logarithm of the rate constant, ^c^ u_eq_—the relative loss of adsorbate from the solution, ^d^ t_1/2_—half-time, ^e^ SD(c)/c_o_—relative standard deviation, ^f^ 1 − R^2^—indetermination coefficient.

**Table 9:** Chosen physicochemical properties of the studied organic compounds [38,39].

Adsorbate	Structural Formula	M ^a^ [g/mol]	c_s_^b^ [g/L]	pKa ^c^	m.p. ^d^ [°C]	b.p. ^e^ [°C]	Chemical Safety
Phenol	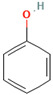	94.11	82.8	9.99	41	182	Corrosive Acute toxic Health hazard
2-Nitrophenol	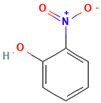	139.11	2.0	7.17	44–46	216	Irritant Environmental hazard
3-Nitrophenol	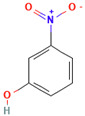	139.11	13.5	8.28	96.8	194	Corrosive Irritant Health hazard
4-Nitrophenol	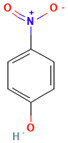	139.11	11.6	7.15	113.8	279	Irritant Health hazard
Methylene blue	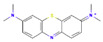	319.85	43.6	>12	100–110	-	Corrosive Irritant

^a^ M—molar mass, ^b^ c_s_—solubility in water at 25 °C, ^c^ pK_a_—ionization constant, ^d^ m.p.—melting point, ^e^ b.p.—boiling point.

## Data Availability

The data and samples are available from the authors.

## Supplementary Materials

The following supporting information can be downloaded at: https://www.mdpi.com/article/10.3390/molecules29092038/s1, Table S1: Relative standard deviations SD(c)/c_o_ (%) for m-exp, FOE, SOE, MOE, f-FOE, f-SOE, f-MOE equations for F, 2-, 3- and 4-NF adsorption kinetics on RIAA activated carbon (constant initial concentration and variable adsorbent mass); Table S2: Relative standard deviations SD(c)/c_o_ (%) for m-exp, FOE, SOE, MOE, f-FOE, f-SOE, f-MOE equations for F, 2-, 3- and 4-NF adsorption kinetics on RIAA activated carbon (constant adsorbent mass and variable initial concentration); Table S3: Relative standard deviations SD(c)/c_o_ (%) for m-exp, FOE, SOE, MOE, f-FOE, f-SOE, f-MOE equations for 4-NF adsorption on on GAC activated carbon at varying pH: 2, 7 and 10; Table S4: Relative standard deviations SD(c)/c_o_ (%) for m-exp, FOE, SOE, MOE, f-FOE, f-SOE, f-MOE equations for MB, 2-, 3- and 4-nitrophenol adsorption from dilute aqueous solutions on GAC activated carbon in single- and multi-component systems: 2-NF+MB, 3-NF+MB and 4-NF+MB.
