# Supplementary material for: Comprehensive Studies of Adsorption Equilibrium and Kinetics for Selected Aromatic Organic Compounds on Activated Carbon
Source: Molecules. 2024 Apr 28;29(9):2038. doi: 10.3390/molecules29092038 (PMC11085397; doi:10.3390/molecules29092038)
Supplement: Supplementary file 1 [file molecules-29-02038-s001.zip › molecules-2942744-supplementary.pdf]

Supplementary material for the paper:

Comprehensive studies of adsorption equilibrium and kinetics for selected aromatic organic compounds on activated carbon

Małgorzata Wasilewska*, Anna Derylo-Marczewska* and Adam W. Marczewski

Department of Physical Chemistry, Institute of Chemical Sciences, Faculty of Chemistry, Maria Curie-Sklodowska University in Lublin, Maria Curie-Sklodowska Sq. 3, 20-031 Lublin, Poland

***Corresponding authors:** e-mail: malgorzata.wasilewska@mail.umcs.pl, anna.derylo-marczewska@mail.umcs.pl

2.2.3. Effect of adsorbent mass

Table S1. Relative standard deviations SD(c)/c_0 (%) for m-exp, FOE, SOE, MOE, f-FOE, f-SOE, f-MOE equations for F, 2-, 3- and 4-NF adsorption kinetics on RIAA activated carbon (constant initial concentration and variable adsorbent mass).

System	m-exp [%]	FOE [%]	SOE [%]	MOE [%]	f-FOE [%]	f-SOE [%]	f-MOE [%]
F/RIAA Co=1.4mM, m=0.05g	0.163	1.650	1.519	1.238	1.396	0.802	0.806
F/RIAA Co=1.4mM, m=0.1g	0.190	2.729	0.929	0.694	1.453	0.194	0.195
F/RIAA Co=1.4mM, m=0.15g	0.832	0.885	2.825	0.843	0.871	0.849	0.797
F/RIAA Co=1.4mM, m=0.2g	0.094	1.302	2.618	0.479	0.705	0.715	0.720
2-NF/RIAA Co=0.323mM, m=0.05g	0.512	3.274	2.861	1.417	1.110	2.303	1.049
2-NF/RIAA Co=0.323mM, m=0.1g	0.344	0.828	5.505	0.357	0.381	2.048	2.059
2-NF/RIAA Co=0.323mM, m=0.15g	0.251	0.398	6.222	0.271	0.315	2.130	16.989
2-NF/RIAA Co=0.323mM, m=0.2g	0.455	0.682	5.721	0.464	0.473	2.204	4.384
3-NF/RIAA Co=0.339mM, m=0.05g	0.246	0.340	5.478	0.303	0.329	1.535	1.534
3-NF/RIAA Co=0.339mM, m=0.1g	0.231	0.234	6.793	0.279	0.427	1.348	1.487
3-NF/RIAA Co=0.339mM, m=0.15g	0.480	0.482	7.066	0.503	0.493	2.197	2.209
3-NF/RIAA Co=0.339mM, m=0.2g	0.451	0.691	5.702	0.442	0.449	2.189	4.369
4-NF/RIAA Co=0.205mM, m=0.05g	0.086	0.430	4.438	0.127	0.525	1.629	1.732

4-NF/RIAA Co=0.205mM, m=0.1g	0.175	0.432	5.785	0.176	0.427	1.495	1.571
4-NF/RIAA Co=0.205mM, m=0.15g	0.327	0.528	6.045	0.364	0.493	2.156	2.189
4-NF/RIAA Co=0.205mM, m=0.2g	0.106	0.589	4.528	0.114	0.461	2.175	4.256

2.2.4. Effect of adsorbate concentrations

Table S2. Relative standard deviations SD(c)/c_o (%) for m-exp, FOE, SOE, MOE, f-FOE, f-SOE, f-MOE equations for F, 2-, 3- and 4-NF adsorption kinetics on RIAA activated carbon (constant adsorbent mass and variable initial concentration).

System	m-exp [%]	FOE [%]	SOE [%]	MOE [%]	f-FOE [%]	f-SOE [%]	f-MOE [%]
F/RIAA Co=1.4mM, m=0.1g	0.190	2.729	0.929	0.694	1.453	0.194	0.195
F/RIAA Co=0.933mM, m=0.1g	0.306	1.942	2.163	1.015	0.887	1.519	1.527
F/RIAA Co=0.7mM, m=0.1g	0.144	1.394	2.210	0.562	2.930	0.543	0.546
F/RIAA Co=467mM, m=0.1g	0.239	0.898	3.450	0.561	0.674	0.911	0.916
2-NF/RIAA Co=0.323mM, m=0.1g	0.344	0.828	5.505	0.357	0.381	2.048	2.059
2-NF/RIAA Co=0.205mM, m=0.1g	0.791	0.792	7.521	0.818	0.817	2.354	0.797
2-NF/RIAA Co=0.161mM, m=0.1g	0.211	0.213	6.887	0.218	0.217	2.310	2.328
2-NF/RIAA Co=0.108mM, m=0.1g	0.556	0.558	6.710	0.621	0.653	1.194	0.639
3-NF/RIAA Co=0.339mM, m=0.1g	0.252	1.068	4.697	0.276	0.449	1.894	16.708
3-NF/RIAA Co=0.205mM, m=0.1g	0.792	0.793	7.511	0.805	0.806	2.334	0.897
3-NF/RIAA Co=0.169mM, m=0.1g	0.211	0.212	6.887	0.314	0.227	2.310	2.328
3-NF/RIAA Co=0.113mM, m=0.1g	0.556	0.558	6.710	0.139	0.634	2.277	2.294
4-NF/RIAA Co=0.205mM, m=0.1g	0.123	0.268	2.681	0.296	0.379	1.685	6.542
4-NF/RIAA Co=0.137mM, m=0.1g	0.382	0.384	5.491	0.385	0.911	1.985	0.769
4-NF/RIAA Co=0.102mM, m=0.1g	0.175	0.178	5.752	0.176	0.427	1.910	2.208
4-NF/RIAA Co=0.068mM, m=0.1g	0.109	0.358	5.680	0.150	0.746	2.189	2.104

2.2.5. Effect of pH

Table S3. Relative standard deviations SD(c)/c_o (%) for m-exp, FOE, SOE, MOE, f-FOE, f-SOE, f-MOE equations for 4-NF adsorption on on GAC activated carbon at varying pH: 2, 7 and 10.

System	m-exp [%]	FOE [%]	SOE [%]	MOE [%]	f-FOE [%]	f-SOE [%]	f-MOE [%]
4-NF/GAC pH=2	0.095	0.133	6.445	0.097	0.349	12.709	0.099
4-NF/GAC pH=7	0.615	0.998	5.248	0.694	0.650	2.163	18.606
4-NF/GAC pH=10	0.434	0.687	5.848	0.543	0.512	2.166	21.564

2.2.6. Effect of the presence of an accompanying substance

Table S4. Relative standard deviations SD(c)/c_o (%) for m-exp, FOE, SOE, MOE, f-FOE, f-SOE, f-MOE equations for MB, 2-, 3- and 4-nitrophenol adsorption from dilute aqueous solutions on GAC activated carbon in single- and multi-component systems: 2-NF+MB, 3-NF+MB and 4-NF+MB.

System	m-exp [%]	FOE [%]	SOE [%]	MOE [%]	f-FOE [%]	f-SOE [%]	f-MOE [%]
2-NF/GAC	0.066	1.021	4.568	0.228	0.397	1.533	0.204
2-NF(2-NF+MB)/GAC	0.769	1.455	3.399	0.835	0.987	1.377	5.386
3-NF/GAC	0.116	0.394	5.936	0.129	0.152	2.043	0.130
3-NF(3-NF+MB)/GAC	0.883	1.208	4.829	0.947	0.963	1.887	5.638
4-NF/GAC	0.095	0.133	6.445	0.097	0.097	0.349	12.709
4-NF(4-NF+MB)/GAC	0.528	0.532	0.668	5.591	0.530	2.037	17.879
MB/GAC	0.513	7.180	15.981	0.673	0.862	1.225	1.228
MB(MB+2-NF)/GAC	0.604	0.610	5.870	0.612	0.607	5.458	5.909
MB(MB+3-NF)/GAC	0.506	0.695	6.429	0.508	0495	2.449	6.182
MB(MB+4-NF)/GAC	0.227	0.826	5.659	0,382	0.331	2.046	2.053
